# Genetic variability of microRNA regulome in human

**DOI:** 10.1002/mgg3.110

**Published:** 2014-09-15

**Authors:** Jana Obsteter, Peter Dovc, Tanja Kunej

**Affiliations:** University of Ljubljana, Biotechnical Faculty, Department of Animal ScienceGroblje 3, 1230, Domzale, Slovenia

**Keywords:** Biogenesis, Dicer, Drosha, human, microRNA (miRNA), polymorphisms

## Abstract

MicroRNAs are currently being extensively studied due to their important role as post-transcriptional regulators. During miRNA biogenesis, precursors undergo two cleavage steps performed by Drosha-DGCR8 (Microprocessor) cleaving of pri-miRNA to produce pre-miRNA and Dicer-mediated cleaving to create mature miRNA. Genetic variants within human miRNA regulome have been shown to influence miRNA expression, target interaction and to affect the phenotype. In this study, we reviewed the literature, existing bioinformatics tools and catalogs associated with polymorphic miRNA regulome, and organized them into four categories: (1) polymorphisms located within miRNA genes (miR-SNPs), (2) transcription factor-binding sites/miRNA regulatory regions (miR-rSNPs), (3) miRNA target sites (miR-TS-SNPs), and 4. miRNA silencing machinery (miR-SM-SNPs). Since the miR-SM-SNPs have not been systematically studied yet, we have collected polymorphisms associated with miRNA silencing machinery. We have developed two catalogs containing genetic variability within: (1) genes encoding three main catalytic components of the silencing machinery, *DROSHA, DGCR8,* and *DICER1*; (2) miRNA genes itself, overlapping Drosha and Dicer cleavage sites. The developed resource of polymorphisms is available online (http://www.integratomics-time.com/miRNA-regulome) and will be useful for further functional studies and development of biomarkers associated with diseases and phenotypic traits.

## Introduction

MicroRNAs (miRNAs) are short noncoding single-stranded RNA molecules, ∼22 nucleotides (nt) in length, which act as post-transcriptional regulators. By binding to the different target gene regions, that is, 3′-untranslated region (3′UTR), 5′UTR, promoter or coding sequences, they repress or activate translation (reviewed in [Kunej et al. 2012]). The crucial binding location for translational regulation resides in the mature miRNA sequence, more accurately within the nucleotides 2–7 or 2–8 from the 5′ end of the miRNA, called the seed region (Sun et al. 2009). During their biogenesis, miRNA undergo many protein interactions, including two catalytic steps performed by two ribonuclease III family enzymes, Drosha and Dicer. Drosha and its cofactor DGCR8 (DiGeorge syndrome Critical Region 8) form a complex called Microprocessor (Gregory et al. 2004), which cleaves pri-miRNA into ∼70-nt-long pre-miRNA. Drosha cleaves on approximately two-thirds of the pri-miRNA stem (Murchison and Hannon 2004). Next step in miRNA biogenesis is catalytic cleavage performed by Dicer enzyme, which cleaves pre-miRNA to create mature miRNA. Catalytic steps, performed by Drosha and Dicer, determine the sequence of mature miRNA. Mature miRNA strand is then loaded into RNA-induced silencing complex (RISC) and participates in the regulation of gene expression.

Georges et al. (2007) made the first effort categorizing miRNA-associated genetic variability and developed bioinformatics tool Patrocles (Hiard et al. 2010) for the search of polymorphisms within miRNA genes, miRNA targets, and genes encoding components of silencing machinery. It has been shown previously that polymorphisms within miRNA genes (miR-SNPs) can influence phenotype and disease development in human and animals (Clop et al. 2006; Sethupathy and Collins 2008). Besides miRNA genes, polymorphisms within miRNA target genes (miR-TS-SNPs) have also been linked to phenotypic changes and diseases (Georges et al. 2007), such as different cancer types (Shao and Brown 2004; Nicoloso et al. 2010; Naccarati et al. 2012; Li et al. 2013; Gong et al. 2014; Mi et al. 2014), Parkinson disease (Wang et al. 2008), asthma (Tan et al. 2007), hypertension (Sethupathy et al. 2007); and disease outcome, such as colorectal adenocarcinoma and Hodgkin lymphoma outcome (Lin et al. 2012; Navarro et al. 2013). Next, polymorphisms within miRNA regulatory regions; variations which affect the ability of a transcription factor to bind to DNA have been shown to influence miRNA expression and were associated with systemic lupus erythematosus (Luo et al. 2011). Polymorphisms related to miRNA silencing machinery have also been investigated for disease association. Some studies indicate the link between polymorphisms within genes encoding components of miRNA silencing machinery and diseases, for example, lung cancer survival (*POLR2A*,*DROSHA*, and *DICER1*) (Rotunno et al. 2010), breast cancer survival (*AGO2*,*DICER1*,*HIWI*,*DGCR8*,*DROSHA*, and *GEMINI4*) (Sung et al. 2012), neck and head cancers (*HIWI, RAN,* and *DICER1*) (Ma et al. 2012), and T-cell lymphoma (*DICER1*) (Li et al. 2012). A recent study demonstrated significant miRNA-SNP-associated changes in Drosha/DGCR8 and/or Dicer processing. Additionally, an association between pre-miRNA SNPs and different mature miRNA levels has also been revealed (Han et al., 2013). Genetic variability associated with miRNA silencing machinery has not been systematically catalogued yet, reviewed and closely examined for common characteristics regarding the effect on miRNA processing. It still remains unclear, how Drosha and Dicer recognize their substrate and choose their cleavage sites, therefore, critical miRNA regions for accurate processing remain undetermined. The lack of this knowledge makes it difficult to understand, which alterations affect miRNA processing – whether they are structural alterations or sequence polymorphisms. Furthermore, Drosha and Dicer enzymes are difficult to obtain or produce. Consequently, the effects of a certain polymorphism on miRNA processing remains poorly investigated.

MicroRNA regulome is the compendium of regulatory elements that either regulate miRNA expression or are regulated by miRNA activity (Bulik-Sullivan et al. 2013). The aim of this study was to: (1) systematically define categories of genetic variability within miRNA regulome and to complement the corresponding nomenclature; (2) collect the existing bioinformatics tools and catalogs associated with polymorphic miRNA regulome, (3) compile and describe genetic variability associated with miRNA silencing machinery in humans. We therefore developed two catalogs comprising genetic variability within: (1) three genes encoding the main catalytic components of the silencing machinery (*DROSHA*,*DGCR8*, and *DICER1*) and (2) miRNA genes overlapping with ±1 nt Drosha and Dicer cleavage sites.

## Material and Methods

### Categorization of polymorphisms within miRNA regulome and review of bioinformatics tools and catalogs

PubMed (http://www.ncbi.nlm.nih.gov/pubmed/) and Web of Science (http://apps.webofknowledge.com/) were used for collecting publications, bioinformatics tools and catalogs regarding miRNA-associated polymorphisms. The search for articles and publications associated with miRNA biogenesis, silencing machinery, and polymorphic miRNA regulome was performed using the following key words: miRNA biogenesis, miRNA silencing machinery, polymorphic miRNA, miRNA target sites, regulatory regions, silencing machinery, miRNA-associated polymorphisms, mutations, genetic variants, and SNPs. Bioinformatics tools and databases developed in support of research of genetic variability within miRNA regulome were collected using the following key words: microRNA, genetic variability, regulatory regions, miRNA target sites, silencing machinery, SNPs, genetic, variants, polymorphisms, and mutations. We retrieved only bioinformatics tools and catalogs described in scientific papers in journals being referred by ISI.

### Catalog of genetic variability within *DROSHA, DGCR8* and *DICER1* genes

GenBank (http://www.ncbi.nlm.nih.gov/genbank/), release 197, was used for nucleotide sequence extraction and Ensembl GRCh37, release 75, database (http://www.ensembl.org/) was used for extracting genetic variability of *DROSHA* (RefSeq NM_013235.4)*, DGCR8* (RefSeq NM_022720.6)*,* and *DICER1* (RefSeq NM_177438.2) genes. Information regarding minor allele frequency (MAF) was obtained from dbSNP, release Human Build 141 (http://www.ncbi.nlm.nih.gov/SNP/). Nonsynonymous polymorphisms with predicted deleterious effect on protein function (SIFT (Sorting Intolerant From Tolerant) value under 0.05) were considered. Uniprot, release 2013_08 (http://www.uniprot.org/), was used to identify functional protein domains within Drosha, DGCR8 and Dicer protein. Each genetic variant in the catalog is provided with polymorphism ID (rs number, COSMIC (Catalogue Of Somatic Mutation In Cancer) name or ESP (Exome Sequencing Project) name), nucleotide variation, MAF, amino acid variation, amino acids coordinates, source and evidence of polymorphism, SIFT value, and domain, within which the polymorphism is located.

### Catalog of genetic variability in miRNA genes overlapping with Drosha or Dicer cleavage sites

The list of miRNAs was obtained using miRBase (http://www.mirbase.org/), release 19. MicroRNA host genes were identified using Ensembl GRCh37, release 74, and miRNA host gene project (Godnic et al. 2013). SNiPer 3.0 tool (http://www.integratomics-time.com/miRNA-SNiPer/ [Jevsinek Skok et al. 2013]) was used to search for polymorphisms residing within ±1 nt of pri-miRNA/pre-miRNA and pre-miRNA/mature miRNA border. Minor allele frequencies were obtained from dbSNP, release Human Build 141 (http://www.ncbi.nlm.nih.gov/SNP/). Each genetic variant was supplemented with information about polymorphic miRNA, miRNA host gene, miRNA strand, position according to the pri-miRNA/pre-miRNA and pre-miRNA mature miRNA (±1 nt), polymorphism ID (rs number, COSMIC name), type of polymorphism (nucleotide variation), validation status, MAF, and the reference, if the polymorphism was previously analyzed. Case of two or more consecutive SNPs within the same miRNA were designated as MNP (multiple nucleotide polymorphism), either DNP (dinucleotide polymorphisms) or TNP (triple nucleotide polymorphisms).

## Results

**Figure 1:**
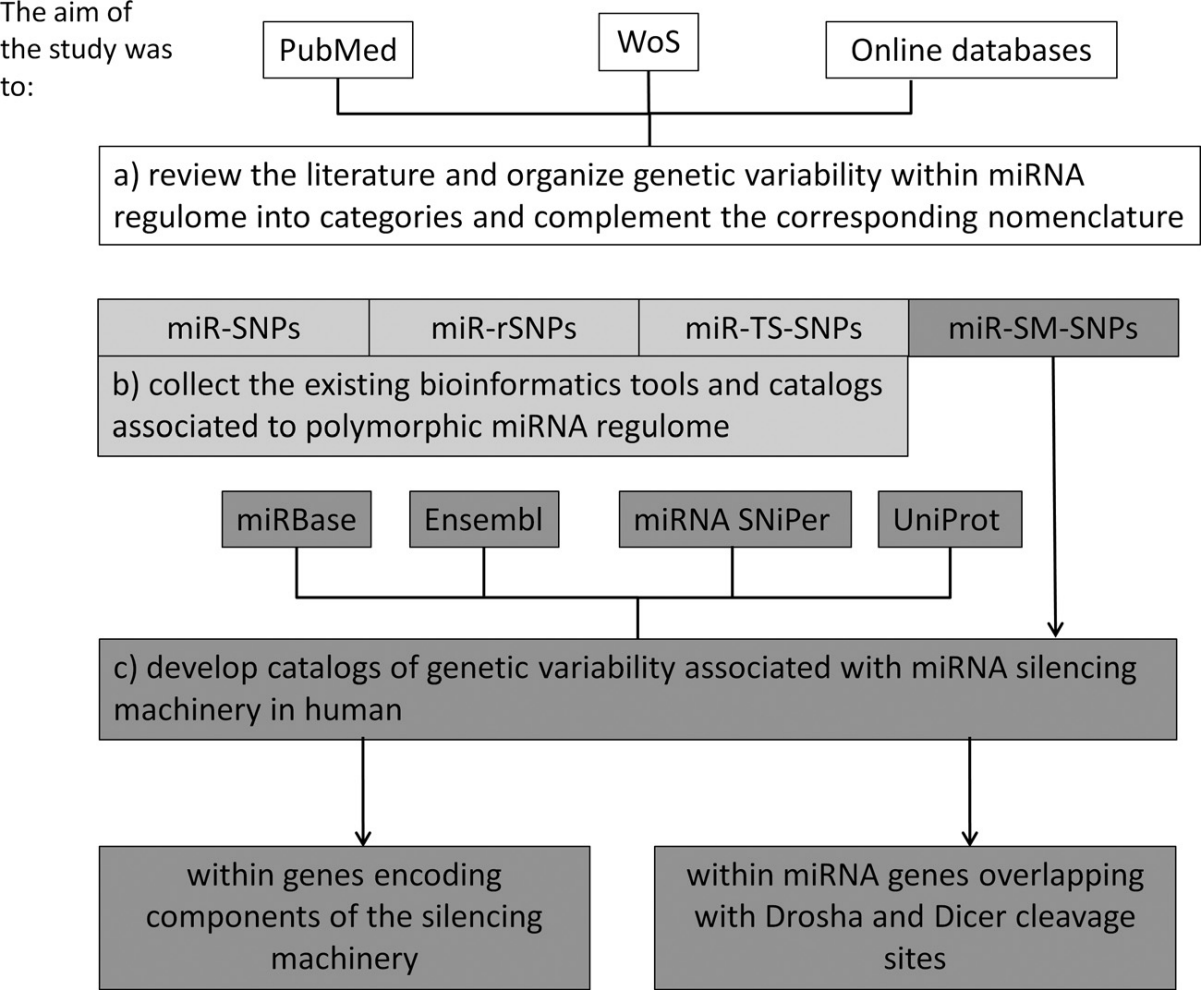
Flowchart of study assembly and aims of the study.

In this study, we systematically integrated and organized information and the nomenclature related to polymorphisms within miRNA regulome, therefore we have reviewed the literature, existing databases and bioinformatics tools and developed new catalogs related to polymorphic silencing machinery (Fig.1).

### Categorization of polymorphisms within miRNA regulome and review of bioinformatics tools and catalogs

**Figure 2:**
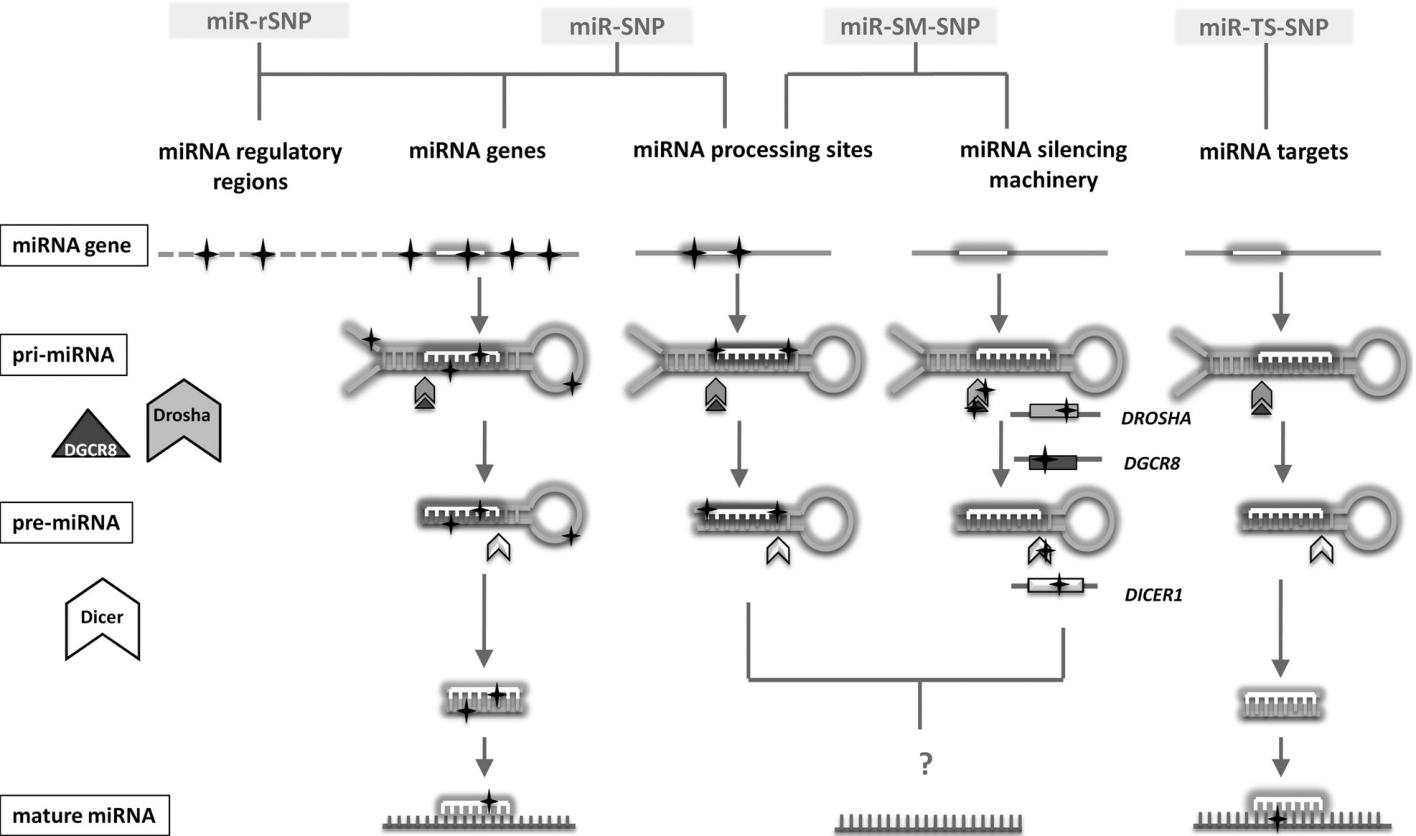
Categories of genetic variability associated with miRNAs.

Based on current knowledge, we organized miRNA-associated genetic variability into four categories: 1. miR-SNPs, 2. miR-rSNPs (miRNA regulatory SNPs), 3. miR-TS-SNPs, and 4. miR-SM-SNPs (miRNA silencing machinery polymorphisms) (Fig.2). If available, we supplemented the categories with the lists of existing bioinformatics tools and catalogs developed in support of research of miRNA genetic variability. The review comprises bioinformatics tools for all four categories (Hariharan et al. 2009; Barenboim et al. 2010; Hiard et al. 2010; Bhartiya et al. 2011; Schmeier et al. 2011; Thomas et al. 2011; Bruno et al. 2012; Gong et al. 2012; Liu et al. 2012; Zorc et al. 2012; Bhattacharya et al. 2013a,b; Deveci et al. 2014). Up to date there are catalogs available only for three categories of miRNA associated polymorphisms: miR-SNPs, miR-SM-SNPs, and miR-TS-SNPs (Landi et al. 2008; Zorc et al. 2012; Jevsinek Skok et al. 2013) (Table S1), but none for miR-rSNPs. All the listed tools and catalogs are supplemented with the information regarding the latest release and the source databases.

### Catalogs compiling polymorphisms associated with miRNA silencing machinery

Since miR-SM-SNPs have not been systematically collected, we developed two new catalogs, compiling polymorphisms associated with miRNA silencing machinery and supplemented it with information relevant to further functional analysis. The catalogs contain: (1) nonsynonymous polymorphisms within *DROSHA*,*DGCR8*, and *DICER1* genes, and (2) polymorphisms within miRNA genes overlapping Drosha or Dicer cleavage site. Both catalogs are freely accessible at URL: http://www.integratomics-time.com/miRNA-regulome and described in the next two paragraphs.

#### Catalog of polymorphisms residing within genes encoding the components of the silencing machinery

**Figure 3:**
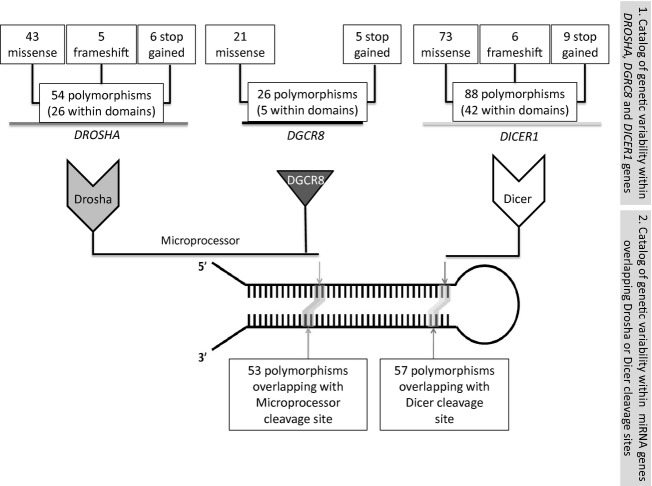
The number of nonsynonymous polymorphisms within genes encoding silencing machinery (*DROSHA, DGCR8, DICER1)* and the number of polymorphisms within Drosha and Dicer cleavage sites.

During their biogenesis, miRNAs undergo two catalytic steps, performed by Microprocessor (Drosha + DGCR8) and Dicer enzyme. Therefore, a catalog compiling polymorphisms in genes encoding three main catalytic components in miRNA biogenesis pathway was created (Fig.3): *DROSHA, DGCR8,* and *DICER1* gene (Tables S2-S4). In this preliminary study, we focused to nonsynonymous polymorphisms, therefore the catalog contains missense, frameshift and stop gained variants: 54 within *DROSHA* gene, 26 within *DGCR8* gene, and 88 genetic variants within *DICER1* gene. The vast majority of polymorphisms comprise one nucleotide: single- nucleotide substitutions and indels. Two of the collected genetic variants comprise more than one nucleotide: double-nucleotide substitution resulting in stop gained codon (*DROSHA* gene) and ten nucleotide frameshift deletions (*DICER1* gene). Furthermore, some of the catalogued nonsynonymous polymorphisms are located within gene regions that correspond to functional protein domains: 26 out of 54 in *DROSHA* gene (Fig. S1A), five out of 26 in *DGCR8* gene (Fig. S1B), and 42 out of 88 in *DICER1* gene (Fig. S1C). The developed catalog could now be further supplemented with data related to polymorphisms within genes encoding other components of miRNA biogenesis pathway (for example Exp5, Ago1, Ago2, Ago3, Ago4, TRBP (TAR RNA binding protein)) and also with other types of polymorphisms like synonymous and intronic polymorphisms.

#### Catalog of genetic variability residing within miRNA genes overlapping Drosha and Dicer cleavage sites

**Figure 4:**
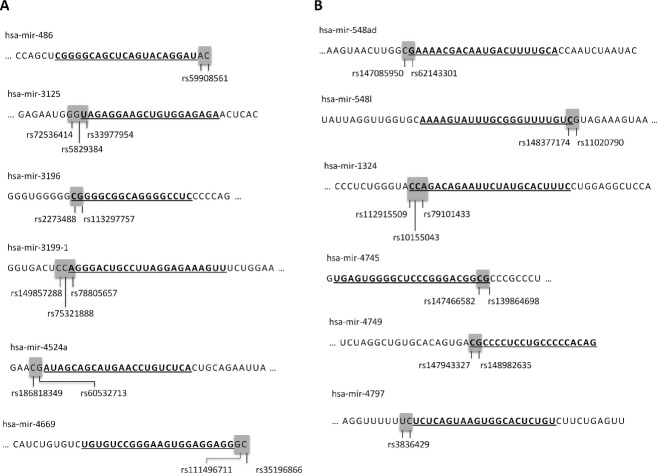
Nucleotide sequences of miRNA genes containing MNPs (DNP or TNP) overlapping Drosha (A) or Dicer (B) cleavage sites.

**Figure 5:**
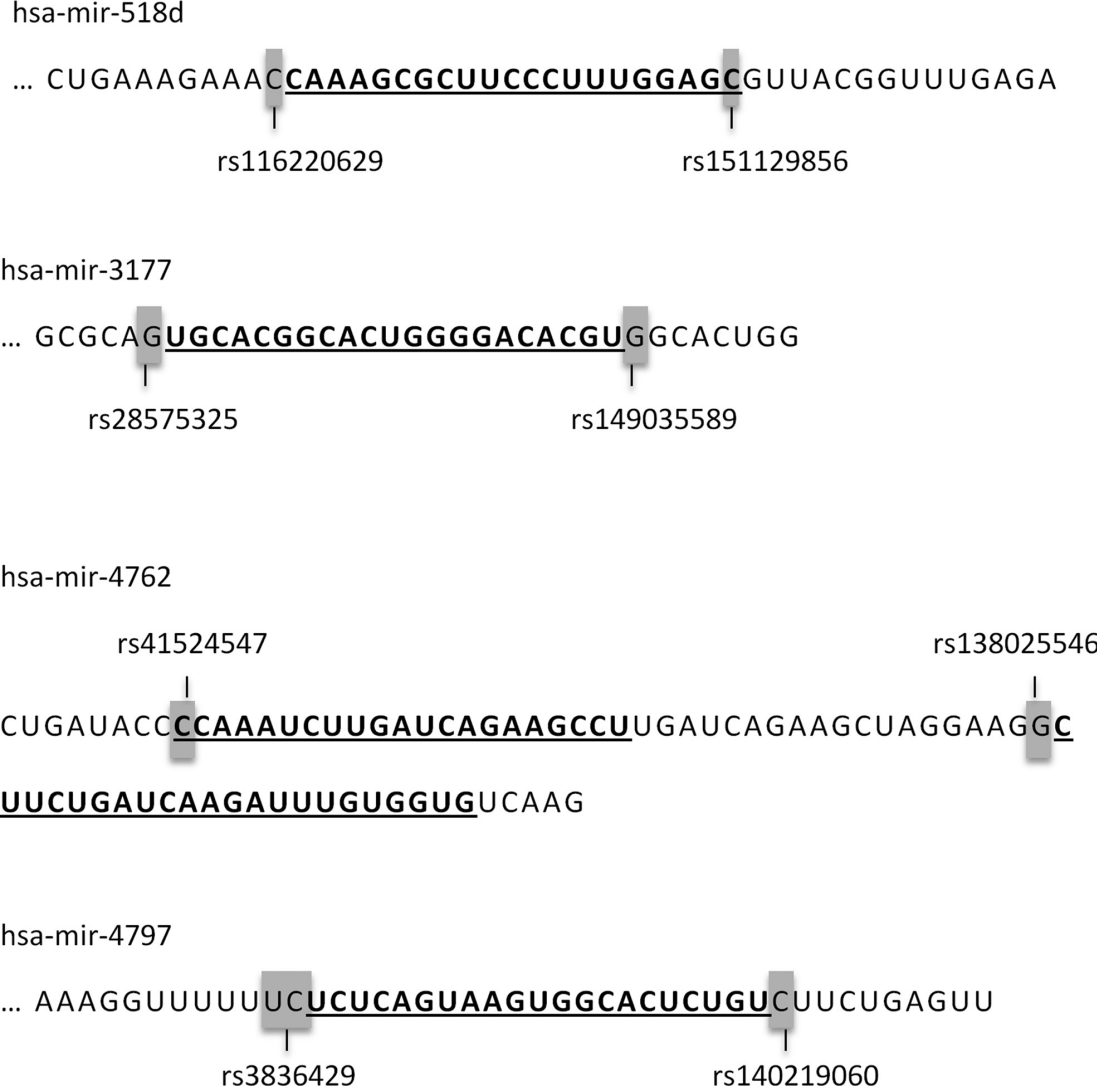
MicroRNA genes with polymorphisms overlapping both, Drosha and Dicer cleavage sites.

Cleavage steps performed by Drosha and Dicer enzymes determine mature miRNA sequence, therefore the catalog of genetic variability residing within miRNA genes overlapping Drosha and Dicer pri- and pre-miRNA and their respective cleavage sites was created (Table S5). In this study, we collected the two adjacent nucleotides according to the pri-miRNA/pre-miRNA border regarding the Drosha cleavage site and the two adjacent nucleotides according to the pre-miRNA/mature miRNA border regarding the Dicer cleavage site. The catalog contains 110 polymorphisms: 53 polymorphisms located within ±1nt Drosha cleavage site and 57 polymorphisms within ±1 nt Dicer cleavage site (Fig.3). Collected polymorphisms comprise single- or multiple-nucleotide substitutions, one or two nucleotide indels, and larger deletions encompassing up to 18 nt. There are six MNPs overlapping the Drosha cleavage site (Fig.4A): four DNPs and two TNPs. Six MNPs were found to overlap the Dicer cleavage site (Fig.4B): five DNPs and one TNP. Three out of 110 polymorphisms have been previously analyzed in experiments and associated with a particular disease – Wilm's tumor (Drake et al. 2009), disease outcome in nasopharyngeal carcinoma (Zheng et al. 2013) or altered miRNA function (Bhattacharya et al. 2012). However, the fact that these polymorphisms are located within ±1 nt from the Drosha or Dicer cleavage site was not the focus of these studies. Our study revealed that there are four miRNA genes containing polymorphisms overlapping both the Drosha and Dicer cleavage site (1 nt): hsa-mir-518d, hsa-mir-3177, hsa-mir-4762, and hsa-mir-4797 (Fig.5). The majority of collected miRNA genes (83/110) was found to be located within the host gene in a sense orientation. Interestingly, hsa-mir-4649 polymorphisms rs113545244 residing within the Drosha cleavage site is also a missense SNP residing within the exon 1 of the *AEBP1* (Adipocyte Enhancer Binding Protein 1) host gene (Fig. S2).

## Discussion

With the aim to systematically describe the polymorphic miRNA regulome, we reviewed existing studies investigating miRNA-associated genetic variability and organized polymorphisms within miRNA regulome into four categories. To facilitate researchers' work we collected existing publicly available bioinformatics tools and catalogs regarding all categories. Additionally, to supplement less explored categories so far, we developed two catalogs containing genetic variants related to miRNA silencing machinery.

First we reviewed literature, databases and bioinformatics tools in order to collect up-to-date knowledge and organized genetic variability into four categories: miR-SNPs, miR-rSNPs, miR-TS-SNPs, and miR-SM-SNPs. Next, we collected previously developed tools and catalogs created to overview and search for polymorphic miRNA regulome genes. While there are many bioinformatics tools developed for identification and analysis of miR-SNPs and miR-TS-SNPs, to our knowledge, there are only two tools existing for analysis of the miR-rSNPs and miR-SM-SNPs. Only a limited number of catalogs compiling polymorphisms within miRNA regulome have been developed so far (Landi et al. 2008; Zorc et al. 2012; Jevsinek Skok et al. 2013), which also became quickly outdated due to frequent updates of genomic databases. Our study is the first review of existing catalogs and tools regarding polymorphic miRNA regulome thus facilitating research work, enabling researchers to easily find the suitable tool or catalog for their study, while Table S1 provides additional information describing content and options of each listed tool or catalog. The review is also the first study presenting the miRNA silencing machinery-related catalogs.

Our first developed catalog contains nonsynonymous genetic variants within genes encoding three components of miRNA biogenesis pathway– *DROSHA, DGCR8,* and *DICER 1* genes. In this preliminary study, three genes were chosen for analysis due to their major role in mature miRNA sequence formation, since Drosha and Dicer enzyme perform two catalytic steps, cleaving on both ends of the mature miRNA sequence. Some of the collected *DROSHA, DGCR8,* and *DICER1* gene polymorphisms are located within regions that correspond to protein domains (Fig. S1). Drosha domains contribute to efficient miRNA processing by performing accurate miRNA binding, cleavage, and DGCR8 binding (Lee et al. 2006), DGCR8 domains are crucial, especially for pri-miRNA binding and substrate specificity of Microprocessor (Yeom et al. 2006), and Dicer domains contribute to pre-miRNA recognition, binding, unwinding, and cleavage (MacRae et al. 2007). According to the given domain function, the effect of the polymorphism on protein function could be predicted. This catalog provides up-to-date information integrated from latest databases and could be useful in studies investigating and determining the function of a particular domain within each of the investigated proteins. Recent findings suggest the importance of synonymous polymorphisms on protein function as well (Hunt et al. 2009), therefore in the future, other than just nonsynonymous genetic variants should be added to the catalog. According to previous studies, polymorphisms located within introns (Rotunno et al. 2010) and 3′UTR or 5′UTR regions (Horikawa et al. 2008) of genes encoding miRNA silencing machinery can also affect miRNA processing. Therefore, polymorphisms in these regions should also be considered for determination of effect on Drosha, DGCR8 or Dicer performance. Additionally, the catalog should also be complemented with polymorphisms within other genes encoding for components of miRNA biogenesis pathway, namely currently there are 52 genes known to be involved in the miRNA silencing process (Hiard et al. 2010). For example, Exportin5 transports pre-miRNA substrates from nucleus to cytoplasm, and Argonaut proteins, specifically Ago2 should also be explored due to their role in RISC assembly and loading. Genes encoding components of the miRNA silencing machinery (*DROSHA, DGCR8,* and *SND1* gene) also host miRNA genes in human (Godnic et al. 2013), therefore it would be interesting to investigate the effect of polymorphisms within these miRNA/host gene pairs on miRNA production and corresponding protein function. The first database compiling miR-SM-SNPs was Patrocles, which includes 52 components of the silencing machinery in seven vertebrate species (Hiard et al. 2010). These genes have been investigated for their genetic variability, overlap with copy number variants as well as eQTL in human or with genes subjected to allelic imbalance.

Additionally, naturally occurring polymorphisms within miRNA genes overlapping Drosha and Dicer cleavage sites could also affect miRNA processing. The developed catalog provides a list of polymorphisms, which may potentially affect Drosha and Dicer cleavage sites, based on their location within 1 nt from the pri-miRNA/pre-miRNA border and the pre-miRNA/mature miRNA border, respectively. Due to the lack of knowledge in this area of research and undetermined Drosha and Dicer substrate recognition mechanisms, it is not clear if only these nucleotides influence accurate pri- and pre-miRNA cleavage. We highlighted polymorphisms possibly having a larger impact on miRNA processing: MNPs within cleavage sites, comprising several consecutive SNPs, and polymorphisms located within exons of miRNA host genes, overlapping Drosha or Dicer cleavage site, which could alter host–gene corresponding protein function as well. MNPs were presented in detail due to the fact that miRNA seed polymorphisms in vertebrates include a high number of DNPs (Zorc et al. 2012; Jevsinek Skok et al. 2013). However, we have to note that the MAFs of these MNPs are mainly very low, except in case of two consecutive SNPs located within hsa-mir-4797, both with MAF of 39%. We also highlighted miRNAs with polymorphisms overlapping both the Drosha and Dicer cleavage site, which could represent useful wild-type models (avoiding introducing mutations) for the study of polymorphisms. However, some of the collected polymorphisms still have unvalidated status; currently, 91/110 polymorphisms have been validated, therefore, some of the collected genetic variants may be sequencing errors. The catalog represents a tool, which could help to determine the function and importance of nucleotides and intact sequence located within Drosha and Dicer cleavage sites, and could be useful in efforts to determine true Drosha and Dicer recognition sites. Our list of genetic variants represents new resource of polymorphisms related to miRNA silencing machinery. With new discoveries regarding Drosha and Dicer recognition and cleavage sites, the catalog could be supplemented with other polymorphisms, crucial for accurate miRNA processing, located outside the reviewed four nucleotides.

In conclusion, the review of polymorphisms within miRNA regulome, bioinformatics tools, and catalogs, including newly developed catalogs associated with miR-SM-SNPs, will be useful in studies investigating the effect of polymorphisms on the functioning of the miRNA silencing machinery. The catalogs provide systematically organized and integrated information about polymorphisms with potential effect on miRNA processing. The collected genes and proteins provide wild type models for researches, allowing them to avoid introducing mutations. Investigation of *DROSHA*,*DGCR8*, and *DICER1* gene polymorphisms could help to determine the key protein residues for accurate enzyme performance and link them with phenotypic traits. Exploring polymorphisms within miRNA genes overlapping Drosha and Dicer cleavage sites could lead to conclusions about the importance of the miRNA sequence and structure at cleavage sites for miRNA processing. It could also help to answer the question, whether the recognition and the cleavage site coincide and which of these sites is crucial for Drosha and Dicer cleavage site determination and enzyme performance. Therefore, both created catalogs will be useful for further functional studies and development of biomarkers associated with diseases and phenotypic traits.

## Supporting Information

Figure S1 Domain structure of Drosha (A), DGCR8 (B) and Dicer (C) protein and nonsynonymous polymorphisms located within their functional domains.

Figure S2 Polymorphism rs113545244 located within hsa-mir-4649 and exon 1 of the *AEBP1* (Adipocyte Enhancer Binding Protein 1) host gene.

Table S1 A review of existing bioinformatics tools and catalogs related to polymorphic miRNA regulome.

Table S2 Catalog of genetic variability residing within *DROSHA* gene.

Table S3 Catalog of genetic variability residing within *DGCR8* gene.

Table S4 Catalog of genetic variability residing within *DICER1* gene.

Table S5 Catalog of genetic variability residing within Drosha and Dicer cleavage sites.
