# Supplementary figures and images for: Genetic variability of microRNA regulome in human
Source: Mol Genet Genomic Med. 2014 Sep 15;3(1):30–9. doi: 10.1002/mgg3.110 (PMC4299713; doi:10.1002/mgg3.110)

## A DROSHA

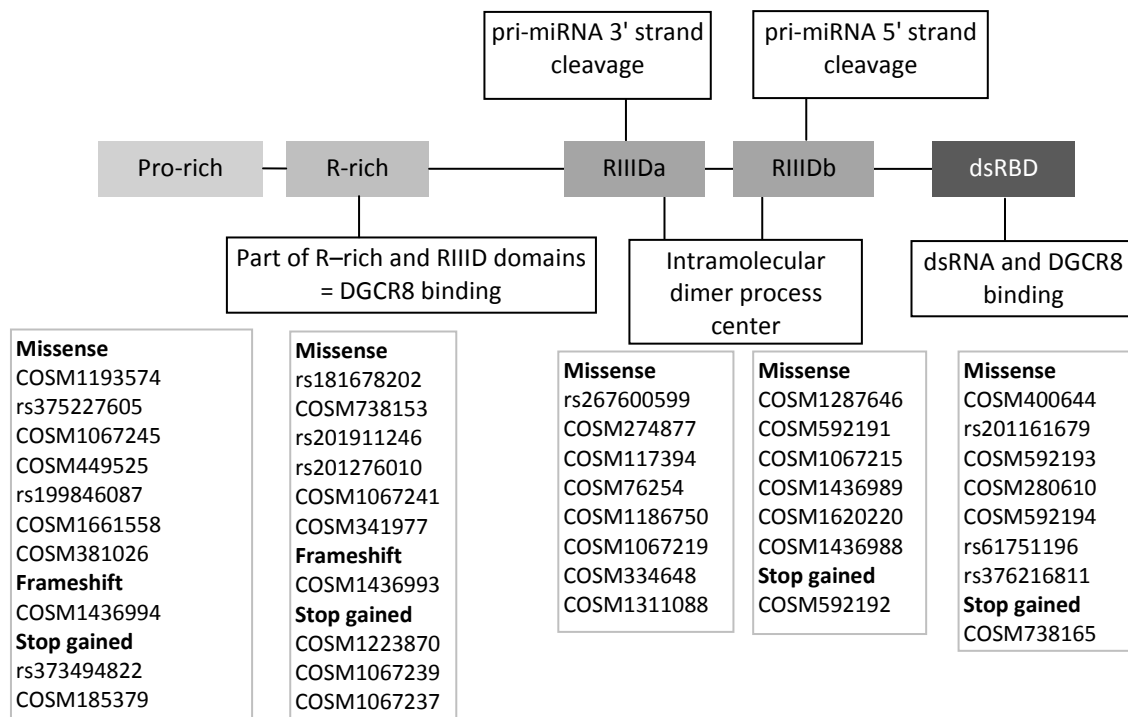

## B DGCR8

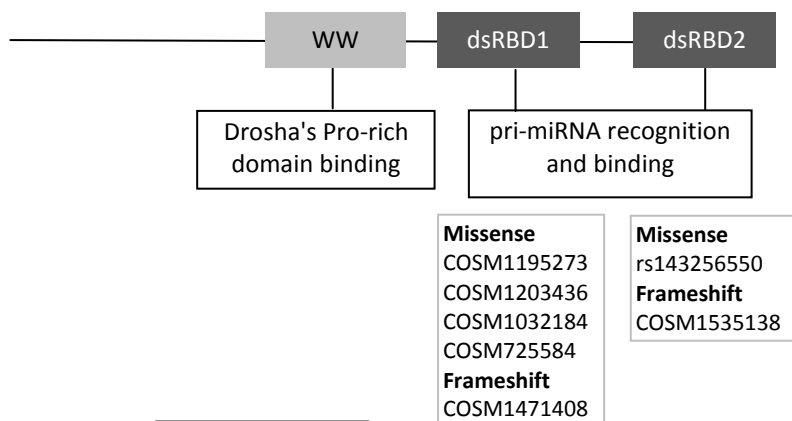

## C DICER

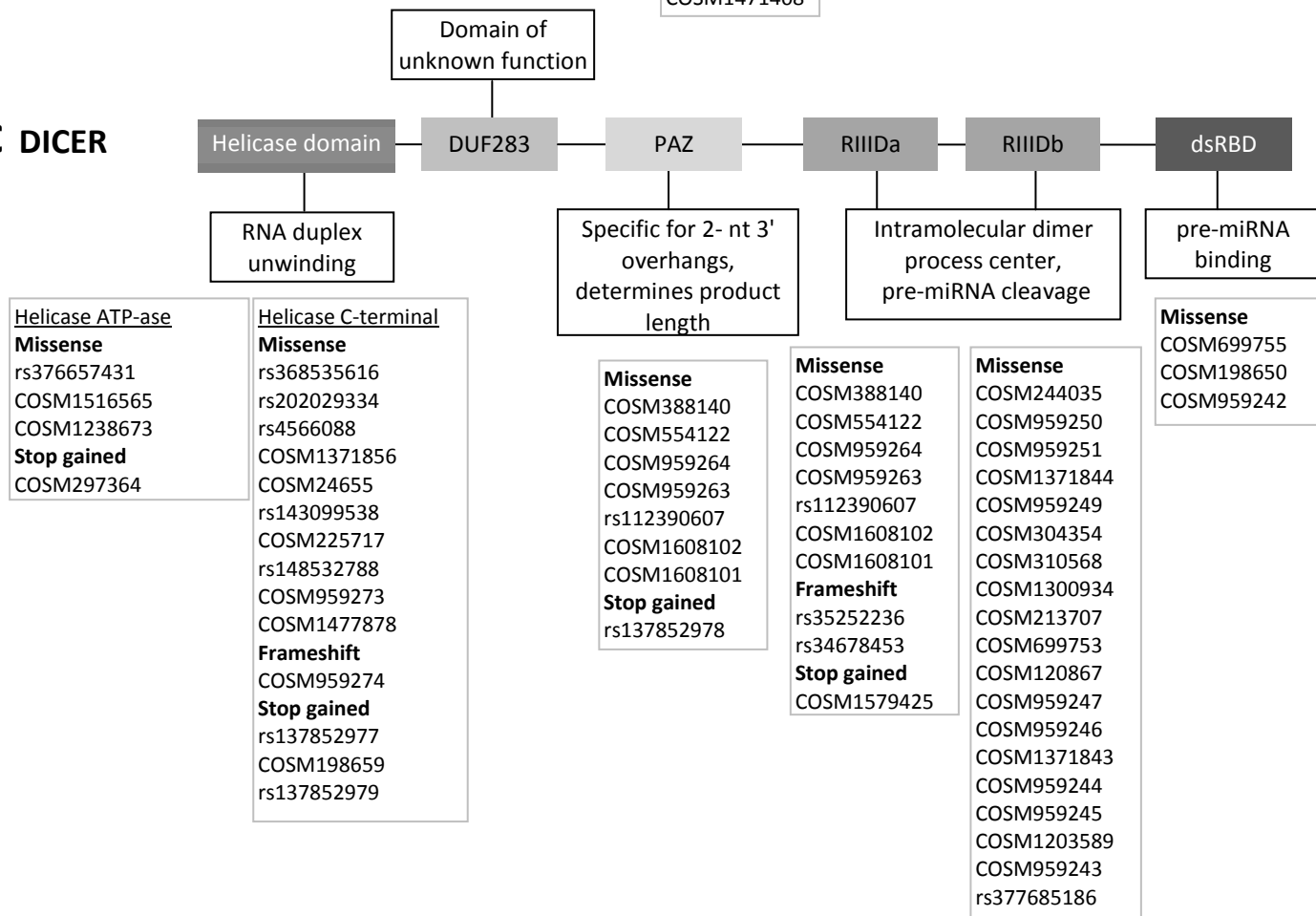

Supplement: Figure S1 — Domain structure of Drosha (A), DGCR8 (B) and Dicer (C) protein and nonsynonymous polymorphisms located within their functional domains. [file mgg30003-0030-sd1.pdf]

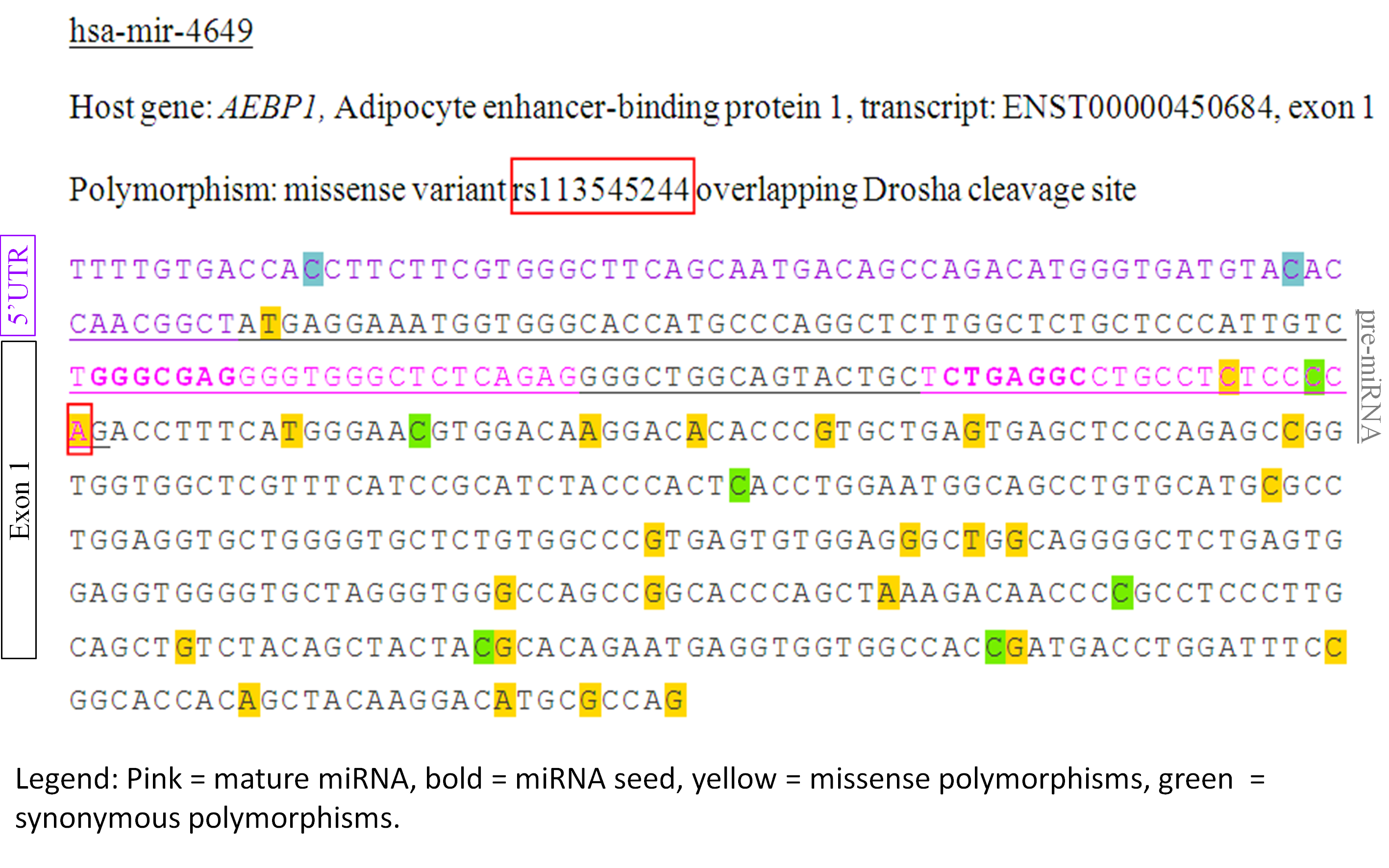

Supplement: Figure S2 — Polymorphism rs113545244 located within hsa-mir-4649 and exon 1 of the AEBP1 (Adipocyte Enhancer Binding Protein 1) host gene. [file mgg30003-0030-sd2.tif]
