# Supplementary material for: Genetic variability of microRNA regulome in human
Source: Mol Genet Genomic Med. 2014 Sep 15;3(1):30–9. doi: 10.1002/mgg3.110 (PMC4299713; doi:10.1002/mgg3.110)
Supplement: Table S1 — A review of existing bioinformatics tools and catalogs related to polymorphic miRNA regulome. [file mgg30003-0030-sd3.docx]

| Polymorphisms within miRNA genes (miR-SNPs)  **Table S1:** A review of existing bioinformatics tools and catalogs related to polymorphic miRNA regulome. | Polymorphisms within miRNA regulatory regions (miR-rSNPs) | Polymorphisms within miRNA target sites (miR-TS-SNPs) | Polymorphisms related to miRNA silencing machinery (miR-SM-SNPs) |
| --- | --- | --- | --- |
| **Tools** | | | |
| miRNA SNP (Gong *et al*., 2012)  <http://www.bioguo.org/miRNASNP/>  The database provides information about SNPs within five major modules: SNPs in human pre-miRNA, SNPs in human miRNA flanks (1kb) (both sorted according to chromosome located on), SNPs in miRNAs of other species (chimpanzee, mouse, rat, dog, horse, cow, chicken, zebrafish), targets gain/loss by SNP in miRNA seed, and targets gain/loss by SNP in target 3’UTR. The tool miRNA SNP v2.0 enables the prediction of the impact of SNPs in genes and miRNA seed regions on miRNA-mRNA interaction and the impact of a SNP on pre-miRNA structure.  *miRNASNP 2.0 (July 2013): miRBase 19 and dbSNP 137. | dPORE – miRNA (Schmeier *et al.,* 2011)  <http://cbrc.kaust.edu.sa/dpore/index.php>  The database integrates information from promoter regions of human miRNA genes, SNPs, and predicted transcription factor binding sites in the promoter regions. It allows search by miRNA (ID, disease, target, pathway), by SNPs (ID, region), or by TF (name).  *[dbSNP130](http://www.ncbi.nlm.nih.gov/projects/SNP/snp_summary.cgi?build_id=130), UCSC hg18, and [Biobase BK](http://www.biobase-international.com/index.php?id=469). | mirdSNP (Bruno *et al.,* 2012)  <http://mirdsnp.ccr.buffalo.edu/index.php>  A database of dSNPs (disease associated SNPs) in human 3’UTR regions. The tool displays relationship between dSNPs, miRNA target site, and SNPs, allowing search by gene name, miRBase ID, target prediction algorithm, disease, and any nucleotide distance between miRNA target site and dSNP.  *UCSC hg18, dbSNP130, HapMap 27,. | Patrocles (Hiard *et al.*, 2010)  <http://www.patrocles.org/>  The database compiles DNA sequence polymorphisms predicted to affect miRNA-mediated gene regulation. Database covers seven vertebrate species and three compartments of silencing process (miRNA precursors, target, and silencing machinery).  *Ensembl 49 and miRBase 11. |
| miRNA SNiPer (Zorc *et al.,* 2012)  <http://www.integratomics-time.com/miRNA-SNiPer/>  The tool allows the search of polymorphisms residing within miRNA genes in 16 species: human, mouse, chicken, chimpanzee, cow, fruit fly, horse, macaque, opossum, orangutan, pig, platypus, rat, tetraodon, zebra finch and zebrafish. It also provides information whether the polymorphism is located within pre-miRNA, miRNA mature, or miRNA mature seed region.  *miRNA-SNiPer 3.0: Ensembl 68 (pig data on 66), miRBase 19, and TargetScan 6.2 |  | MicroSNiPer (Barenboim *et al.,* 2010)  <http://cbdb.nimh.nih.gov/microsniper/>  This application predicts if a SNP within 3’UTR regions of putative miRNA target site will disrupt/eliminate, enhance/create miRNA binding site. MicroSNiPer examines the effect of SNP in real time.  *dbSNP 29; miRBase 13.0, HapMap project (2007) |  |
| SomamiR DB (Bhattacharya *et al.,* 2013b)  <http://compbio.uthsc.edu/SomamiR/>  The database includes information about SNPs miRNA-related mutations in cancer, located within 3’UTR regions of miRNA target sites or within miRNA genes. SomamiR database can be browsed according to somatic mutations that alter miRNA target sites, impact of miRNA related somatic mutations in cancer pathways, genes associated with cancer risk that contain miRNA related somatic mutations, somatic mutations in miRNA sequences.  *UCSC hg19, miRBase 17, and TargetScan 6.0, COSMIC, PCGP, ICGC. |  | MiRSNP (Liu *et al.,* 2012)  <http://202.38.126.151/hmdd/mirsnp/search/>  The database collects of human SNPs in predicted miRNA-mRNA binding sites. SNPs have been annotated for prediction, whether a particular SNP would decrease/break or enhance/create miRNA-mRNA binding site. It allows search by gene, mRNA, SNP, or miRNA.  *dbSNP135, miRBase 18, and HapMap project |  |
| miRvar (Bhartiya *et al.,* 2011)  http://genome.igib.res.in/mirlovd  The database provides a curated list of genetic variations within miRNA genes. If available, it also contains the information concerning of the consequence of particular variation: effect on Dicer cleavage (PhDcleav score), effect on RISC binding (Riscbinder effect score), and disease association.  *dbSNP130 (2006), UCSC hg18, and miRBase 13 |  | dbSMR (Hariharan *et al.,* 2009)  <http://miracle.igib.res.in/dbSMR>  The database is a resource of genome-wide SNPs affecting miRNA mediated regulation. It contains polymorphisms within 200 nt around predicted miRNA target sites, possible to alter target site intramolecular structure and later regulation.  *miRBase v9, Ensembl 47 |  |
| PolymiRTS database (Bhattacharya *et al.,* 2013a)  <http://compbio.uthsc.edu/miRSNP/>  Polymorphisms in microRNAs and their target sites. Database compiles naturally occurring polymorphisms in miRNA seed regions and miRNA target sites. It collects information about SNPs and Indels within miRNA targets (from CLASH experiments, other experimental supports, and predicted miRNA targets) and miRNA seed regions, linking it to diseases, traits, and biological pathways.  *latest release 3.0: dbSNP137; UCSC mm10, hg19; miRBase 20, and TargetScan 6.2 |  | SNP effects on microRNA targeting (Thomas *et al.,* 2011)  http://www.bigr.medisin.ntnu.no/mirsnpscore/  This tool provides computer-based predictions of SNP effects on microRNA-based gene regulation and helps with identification of disease associated SNPs. The tool works by predicting the effect of particular SNP in miRNA target site and uses linkage disequilibrium to map this SNP to SNP of interest in GWAS.  *UCSC hg18, mm9, miRBase, 13.0 and 16.0; HapMap 22 for haplotype data and 27 for linkage disequilibrium data |  |
| Patrocles (Hiard *et al.,* 2010)  <http://www.patrocles.org/> |  | mrSNP (Deveci *et al.,* 2014)  http://mrsnp.osu.edu/  Server predicts the effect of SNPs in 3’UTR on miRNA binding. Server mrSNP correctly identified 69 % of the SNPs that disrupt binding. The tool allows screening of 11 species for SNP effect.  *UCSC hg19 |  |
|  |  | miRNA SNP (Gong *et al.,* 2012) |  |
|  | ; | SomamiR DB (Bhattacharya *et al.,* 2013b) |  |
|  |  | Patrocles (Hiard *et al.,* 2010) |  |
|  |  | PolymiRTS database (Bhattacharya *et al.,* 2013a) |  |
| **Catalogs** | | | |
| MicroRNA genetic variability in livestock species (Jevsinek Skok *et al.,* 2013)  Based on: Ensembl Variation database releases 66 and 68, miRBase releases 18 and 19, and TargetScan 6.2. |  | Catalog of polymorphisms falling in microRNA-binding regions of cancer genes (Landi *et al.,* 2008)  Based on: DIANA-MicroT, miRBase, miRanda, MicroInspector, TargetScan, PicTar | This study |
| Mature miRNA seed polymorphisms in vertebrates (Zorc *et al.*, 2012)  Based on: Ensembl Variation database 64, miRBase 18, and TargetScan 5.2 |  |  |  |

*=based on, COSMIC = Catalogue of Somatic Mutations in Cancer, PCGP = Pediatric Cancer Genome Project, ICGC = International Cancer Genome Consortium.
