# Supplementary material for: Genetic variability of microRNA regulome in human
Source: Mol Genet Genomic Med. 2014 Sep 15;3(1):30–9. doi: 10.1002/mgg3.110 (PMC4299713; doi:10.1002/mgg3.110)
Supplement: Table S2 — Catalog of genetic variability residing within DROSHA gene. [file mgg30003-0030-sd4.docx]

Supplementary table 1: Catalog of genetic variability residing within *DROSHA* gene.

| **Polymorphism ID** | **SNP alleles** | **MAF** | **AA**  **alteration** | **AA coordinates** | **Source** | **Evidence** | **SIFT value** | **Domain** |
| --- | --- | --- | --- | --- | --- | --- | --- | --- |
| **Missense** | | | | | | | | |
| [COSM1193574](http://www.ensembl.org/Homo_sapiens/Variation/Mappings?db=core;g=ENSG00000113360;r=5:31400604-31532303;t=ENST00000511367;v=COSM1193574;vf=69127396;source=COSMIC) | C/T | NA | G/E | 21 | COSMIC | Unknown | 0.02 | Pro-rich |
| [rs375227605](http://www.ensembl.org/Homo_sapiens/Variation/Mappings?db=core;g=ENSG00000113360;r=5:31400604-31532303;t=ENST00000511367;v=rs375227605;vf=63428046;source=dbSNP) | G/A | NA | S/L | 30 | dbSNP | ESP | 0.01 | Pro-rich |
| [COSM1067245](http://www.ensembl.org/Homo_sapiens/Variation/Mappings?db=core;g=ENSG00000113360;r=5:31400604-31532303;t=ENST00000511367;v=COSM1067245;vf=69127395;source=COSMIC) | C/A | NA | R/M | 35 | COSMIC | Unknown | 0.01 | Pro-rich |
| [COSM449525](http://www.ensembl.org/Homo_sapiens/Variation/Mappings?db=core;g=ENSG00000113360;r=5:31400604-31532303;t=ENST00000511367;v=COSM449525;vf=69127390;source=COSMIC) | A/C | NA | F/C | 78 | COSMIC | Unknown | 0 | Pro-rich |
| [rs199846087](http://www.ensembl.org/Homo_sapiens/Variation/Mappings?db=core;g=ENSG00000113360;r=5:31400604-31532303;t=ENST00000511367;v=rs199846087;vf=53955561;source=dbSNP) | G/A | NA | P/L | 100 | dbSNP | ESP, MO | 0.04 | Pro-rich |
| [COSM1661558](http://www.ensembl.org/Homo_sapiens/Variation/Mappings?db=core;g=ENSG00000113360;r=5:31400604-31532303;t=ENST00000511367;v=COSM1661558;vf=69127383;source=COSMIC) | G/C | NA | P/R | 130 | COSMIC | Unknown | 0.05 | Pro-rich |
| [COSM381026](http://www.ensembl.org/Homo_sapiens/Variation/Mappings?db=core;g=ENSG00000113360;r=5:31400604-31532303;t=ENST00000511367;v=COSM381026;vf=69127374;source=COSMIC) | G/C | NA | S/C | 173 | COSMIC | Unknown | 0.01 | Pro-rich |
| [rs181678202](http://www.ensembl.org/Homo_sapiens/Variation/Mappings?db=core;g=ENSG00000113360;r=5:31400604-31532303;t=ENST00000511367;v=rs181678202;vf=41982758;source=dbSNP) | T/C | C=0.000/1 | R/G | 232 | dbSNP | 1000 Genomes | 0.01 | R-rich |
| [COSM738153](http://www.ensembl.org/Homo_sapiens/Variation/Mappings?db=core;g=ENSG00000113360;r=5:31400604-31532303;t=ENST00000511367;v=COSM738153;vf=69127370;source=COSMIC) | C/A | NA | R/L | 234 | COSMIC | Unknown | 0.04 | R-rich |
| [rs201911246](http://www.ensembl.org/Homo_sapiens/Variation/Mappings?db=core;g=ENSG00000113360;r=5:31400604-31532303;t=ENST00000511367;v=rs201911246;vf=55897581;source=dbSNP) | G/A | NA | R/W | 264 | dbSNP | ESP, MO | 0 | R-rich |
| [rs201276010](http://www.ensembl.org/Homo_sapiens/Variation/Mappings?db=core;g=ENSG00000113360;r=5:31400604-31532303;t=ENST00000511367;v=rs201276010;vf=55469649;source=dbSNP) | G/A | NA | R/C | 279 | dbSNP | ESP, MO | 0.05 | R-rich |
| [COSM1067241](http://www.ensembl.org/Homo_sapiens/Variation/Mappings?db=core;g=ENSG00000113360;r=5:31400604-31532303;t=ENST00000511367;v=COSM1067241;vf=69127366;source=COSMIC) | G/A | NA | R/C | 279 | COSMIC | Unknown | 0.05 | R-rich |
| [COSM341977](http://www.ensembl.org/Homo_sapiens/Variation/Mappings?db=core;g=ENSG00000113360;r=5:31400604-31532303;t=ENST00000511367;v=COSM341977;vf=69127361;source=COSMIC) | G/C | NA | S/C | 302 | COSMIC | Unknown | 0.01 | R-rich |
| [COSM1067235](http://www.ensembl.org/Homo_sapiens/Variation/Mappings?db=core;g=ENSG00000113360;r=5:31400604-31532303;t=ENST00000511367;v=COSM1067235;vf=69127358;source=COSMIC) | G/T | NA | S/Y | 343 | COSMIC | Unknown | 0.05 | - |
| [rs369352228](http://www.ensembl.org/Homo_sapiens/Variation/Mappings?db=core;g=ENSG00000113360;r=5:31400604-31532303;t=ENST00000511367;v=rs369352228;vf=57997300;source=dbSNP) | G/A | NA | R/C | 354 | dbSNP | ESP | 0.01 | - |
| [rs187981384](http://www.ensembl.org/Homo_sapiens/Variation/Mappings?db=core;g=ENSG00000113360;r=5:31400604-31532303;t=ENST00000511367;v=rs187981384;vf=48313449;source=dbSNP) | C/T | T=0.0009/2 | R/H | 354 | dbSNP | 1000 Genomes | 0.03 | - |
| [COSM1067233](http://www.ensembl.org/Homo_sapiens/Variation/Mappings?db=core;g=ENSG00000113360;r=5:31400604-31532303;t=ENST00000511367;v=COSM1067233;vf=69127356;source=COSMIC) | G/A | NA | R/C | 364 | COSMIC | Unknown | 0 | - |
| [COSM738155](http://www.ensembl.org/Homo_sapiens/Variation/Mappings?db=core;g=ENSG00000113360;r=5:31400604-31532303;t=ENST00000511367;v=COSM738155;vf=69127355;source=COSMIC) | G/T | NA | R/S | 364 | COSMIC | Unknown | 0.05 | - |
| [rs201711846](http://www.ensembl.org/Homo_sapiens/Variation/Mappings?db=core;g=ENSG00000113360;r=5:31400604-31532303;t=ENST00000511367;v=rs201711846;vf=55722226;source=dbSNP) | G/A | NA | R/C | 371 | dbSNP | ESP, MO | 0 | - |
| [rs372990492](http://www.ensembl.org/Homo_sapiens/Variation/Mappings?db=core;g=ENSG00000113360;r=5:31400604-31532303;t=ENST00000511367;v=rs372990492;vf=61350681;source=dbSNP) | C/T | NA | R/H | 371 | dbSNP | ESP | 0.02 | - |
| [COSM399164](http://www.ensembl.org/Homo_sapiens/Variation/Mappings?db=core;g=ENSG00000113360;r=5:31400604-31532303;t=ENST00000511367;v=COSM399164;vf=69127349;source=COSMIC) | G/C | NA | S/C | 433 | COSMIC | Unknown | 0 | - |
| [COSM738157](http://www.ensembl.org/Homo_sapiens/Variation/Mappings?db=core;g=ENSG00000113360;r=5:31400604-31532303;t=ENST00000511367;v=COSM738157;vf=69127345;source=COSMIC) | C/A | NA | G/W | 453 | COSMIC | Unknown | 0.01 | - |
| [rs370207689](http://www.ensembl.org/Homo_sapiens/Variation/Mappings?db=core;g=ENSG00000113360;r=5:31400604-31532303;t=ENST00000511367;v=rs370207689;vf=58736591;source=dbSNP) | G/A | NA | R/C | 510 | dbSNP | ESP | 0.05 | - |
| [COSM1328847](http://www.ensembl.org/Homo_sapiens/Variation/Mappings?db=core;g=ENSG00000113360;r=5:31400604-31532303;t=ENST00000511367;v=COSM1328847;vf=69127338;source=COSMIC) | C/T | NA | R/H | 544 | COSMIC | Unknown | 0.01 | - |
| [rs372281038](http://www.ensembl.org/Homo_sapiens/Variation/Mappings?db=core;g=ENSG00000113360;r=5:31400604-31532303;t=ENST00000511367;v=rs372281038;vf=60735293;source=dbSNP) | C/A | NA | G/V | 569 | dbSNP | ESP | 0.01 | - |
| [COSM1543324](http://www.ensembl.org/Homo_sapiens/Variation/Mappings?db=core;g=ENSG00000113360;r=5:31400604-31532303;t=ENST00000511367;v=COSM1543324;vf=69127336;source=COSMIC) | C/A | NA | R/I | 570 | COSMIC | Unknown | 0.03 | - |
| [COSM1223869](http://www.ensembl.org/Homo_sapiens/Variation/Mappings?db=core;g=ENSG00000113360;r=5:31400604-31532303;t=ENST00000511367;v=COSM1223869;vf=69127332;source=COSMIC) | G/A | NA | H/Y | 609 | COSMIC | Unknown | 0 | - |
| [COSM1067223](http://www.ensembl.org/Homo_sapiens/Variation/Mappings?db=core;g=ENSG00000113360;r=5:31400604-31532303;t=ENST00000511367;v=COSM1067223;vf=69127329;source=COSMIC) | G/C | NA | P/R | 637 | COSMIC | Unknown | 0 | - |
| [COSM185364](http://www.ensembl.org/Homo_sapiens/Variation/Mappings?db=core;g=ENSG00000113360;r=5:31400604-31532303;t=ENST00000511367;v=COSM185364;vf=69127326;source=COSMIC) | G/T | NA | L/I | 703 | COSMIC | Unknown | 0.04 | - |
| [COSM1311089](http://www.ensembl.org/Homo_sapiens/Variation/Mappings?db=core;g=ENSG00000113360;r=5:31400604-31532303;t=ENST00000511367;v=COSM1311089;vf=69127325;source=COSMIC) | A/G | NA | I/T | 719 | COSMIC | Unknown | 0.01 | - |
| [rs12517177](http://www.ensembl.org/Homo_sapiens/Variation/Mappings?db=core;g=ENSG00000113360;r=5:31400604-31532303;t=ENST00000511367;v=rs12517177;vf=8763865;source=dbSNP) | G/C | NA | L/V | 757 | dbSNP | MO | 0.01 | - |
| [COSM482706](http://www.ensembl.org/Homo_sapiens/Variation/Mappings?db=core;g=ENSG00000113360;r=5:31400604-31532303;t=ENST00000511367;v=COSM482706;vf=69127323;source=COSMIC) | C/T | NA | R/H | 759 | COSMIC | Unknown | 0 | - |
| [COSM1436991](http://www.ensembl.org/Homo_sapiens/Variation/Mappings?db=core;g=ENSG00000113360;r=5:31400604-31532303;t=ENST00000511367;v=COSM1436991;vf=69127322;source=COSMIC) | C/A | NA | Q/H | 761 | COSMIC | Unknown | 0.03 | - |
| [COSM738160](http://www.ensembl.org/Homo_sapiens/Variation/Mappings?db=core;g=ENSG00000113360;r=5:31400604-31532303;t=ENST00000511367;v=COSM738160;vf=69127321;source=COSMIC) | G/A | NA | P/S | 770 | COSMIC | Unknown | 0.04 | - |
| [COSM1223867](http://www.ensembl.org/Homo_sapiens/Variation/Mappings?db=core;g=ENSG00000113360;r=5:31400604-31532303;t=ENST00000511367;v=COSM1223867;vf=69127320;source=COSMIC) | G/A | NA | R/C | 778 | COSMIC | Unknown | 0.02 | - |
| [COSM1620221](http://www.ensembl.org/Homo_sapiens/Variation/Mappings?db=core;g=ENSG00000113360;r=5:31400604-31532303;t=ENST00000511367;v=COSM1620221;vf=69127319;source=COSMIC) | C/T | NA | R/H | 778 | COSMIC | Unknown | 0.04 | - |
| [rs376262500](http://www.ensembl.org/Homo_sapiens/Variation/Mappings?db=core;g=ENSG00000113360;r=5:31400604-31532303;t=ENST00000511367;v=rs376262500;vf=64490520;source=dbSNP) | G/A | NA | R/C | 801 | dbSNP | ESP | 0 | - |
| [COSM738161](http://www.ensembl.org/Homo_sapiens/Variation/Mappings?db=core;g=ENSG00000113360;r=5:31400604-31532303;t=ENST00000511367;v=COSM738161;vf=69127318;source=COSMIC) | C/A | NA | K/N | 817 | COSMIC | Unknown | 0 | - |
| [COSM1067221](http://www.ensembl.org/Homo_sapiens/Variation/Mappings?db=core;g=ENSG00000113360;r=5:31400604-31532303;t=ENST00000511367;v=COSM1067221;vf=69127314;source=COSMIC) | G/A | NA | T/M | 839 | COSMIC | Unknown | 0 | - |
| [rs1559205](http://www.ensembl.org/Homo_sapiens/Variation/Mappings?db=core;g=ENSG00000113360;r=5:31400604-31532303;t=ENST00000511367;v=rs1559205;vf=1215375;source=dbSNP) | G/A | NA | S/F | 854 | dbSNP | HapMap, MO | 0.04 | - |
| [rs372313396](http://www.ensembl.org/Homo_sapiens/Variation/Mappings?db=core;g=ENSG00000113360;r=5:31400604-31532303;t=ENST00000511367;v=rs372313396;vf=60786656;source=dbSNP) | G/A | NA | R/C | 871 | dbSNP | ESP | 0 | - |
| [rs267600599](http://www.ensembl.org/Homo_sapiens/Variation/Mappings?db=core;g=ENSG00000113360;r=5:31400604-31532303;t=ENST00000511367;v=rs267600599;vf=56266251;source=dbSNP) | G/A | NA | R/C | 890 | dbSNP | Unknown | 0 | RIIIDa |
| [COSM274877](http://www.ensembl.org/Homo_sapiens/Variation/Mappings?db=core;g=ENSG00000113360;r=5:31400604-31532303;t=ENST00000511367;v=COSM274877;vf=69127312;source=COSMIC) | G/A | NA | R/C | 890 | COSMIC | Unknown | 0 | RIIIDa |
| [COSM117394](http://www.ensembl.org/Homo_sapiens/Variation/Mappings?db=core;g=ENSG00000113360;r=5:31400604-31532303;t=ENST00000511367;v=COSM117394;vf=69127311;source=COSMIC) | T/A | NA | D/V | 929 | COSMIC | Unknown | 0 | RIIIDa |
| [COSM76254](http://www.ensembl.org/Homo_sapiens/Variation/Mappings?db=core;g=ENSG00000113360;r=5:31400604-31532303;t=ENST00000511367;v=COSM76254;vf=69127310;source=COSMIC) | C/G | NA | R/T | 930 | COSMIC | Unknown | 0 | RIIIDa |
| [COSM1186750](http://www.ensembl.org/Homo_sapiens/Variation/Mappings?db=core;g=ENSG00000113360;r=5:31400604-31532303;t=ENST00000511367;v=COSM1186750;vf=69127306;source=COSMIC) | T/C | NA | I/V | 1003 | COSMIC | Unknown | 0.02 | RIIIDa |
| [COSM1067219](http://www.ensembl.org/Homo_sapiens/Variation/Mappings?db=core;g=ENSG00000113360;r=5:31400604-31532303;t=ENST00000511367;v=COSM1067219;vf=69127305;source=COSMIC) | A/C | NA | F/C | 1021 | COSMIC | Unknown | 0 | RIIIDa |
| [COSM334648](http://www.ensembl.org/Homo_sapiens/Variation/Mappings?db=core;g=ENSG00000113360;r=5:31400604-31532303;t=ENST00000511367;v=COSM334648;vf=69127303;source=COSMIC) | C/A | NA | G/V | 1027 | COSMIC | Unknown | 0 | RIIIDa |
| [COSM1311088](http://www.ensembl.org/Homo_sapiens/Variation/Mappings?db=core;g=ENSG00000113360;r=5:31400604-31532303;t=ENST00000511367;v=COSM1311088;vf=69127304;source=COSMIC) | C/T | NA | G/E | 1027 | COSMIC | Unknown | 0.02 | RIIIDa |
| [rs370990538](http://www.ensembl.org/Homo_sapiens/Variation/Mappings?db=core;g=ENSG00000113360;r=5:31400604-31532303;t=ENST00000511367;v=rs370990538;vf=59471798;source=dbSNP) | G/A | NA | R/C | 1066 | dbSNP | ESP | 0.02 | - |
| [COSM1067217](http://www.ensembl.org/Homo_sapiens/Variation/Mappings?db=core;g=ENSG00000113360;r=5:31400604-31532303;t=ENST00000511367;v=COSM1067217;vf=69127299;source=COSMIC) | T/A | NA | N/I | 1080 | COSMIC | Unknown | 0.01 | - |
| [COSM1287646](http://www.ensembl.org/Homo_sapiens/Variation/Mappings?db=core;g=ENSG00000113360;r=5:31400604-31532303;t=ENST00000511367;v=COSM1287646;vf=69127297;source=COSMIC) | G/T | NA | D/E | 1151 | COSMIC | Unknown | 0 | RIIIDb |
| [COSM592191](http://www.ensembl.org/Homo_sapiens/Variation/Mappings?db=core;g=ENSG00000113360;r=5:31400604-31532303;t=ENST00000511367;v=COSM592191;vf=69127296;source=COSMIC) | C/T | NA | M/I | 1154 | COSMIC | Unknown | 0.03 | RIIIDb |
| [COSM1067215](http://www.ensembl.org/Homo_sapiens/Variation/Mappings?db=core;g=ENSG00000113360;r=5:31400604-31532303;t=ENST00000511367;v=COSM1067215;vf=69127295;source=COSMIC) | G/T | NA | H/N | 1169 | COSMIC | Unknown | 0 | RIIIDb |
| [COSM1436989](http://www.ensembl.org/Homo_sapiens/Variation/Mappings?db=core;g=ENSG00000113360;r=5:31400604-31532303;t=ENST00000511367;v=COSM1436989;vf=69127294;source=COSMIC) | T/C | NA | H/R | 1170 | COSMIC | Unknown | 0.01 | RIIIDb |
| [COSM1620220](http://www.ensembl.org/Homo_sapiens/Variation/Mappings?db=core;g=ENSG00000113360;r=5:31400604-31532303;t=ENST00000511367;v=COSM1620220;vf=69127293;source=COSMIC) | C/T | NA | E/K | 1171 | COSMIC | Unknown | 0 | RIIIDb |
| [COSM1436988](http://www.ensembl.org/Homo_sapiens/Variation/Mappings?db=core;g=ENSG00000113360;r=5:31400604-31532303;t=ENST00000511367;v=COSM1436988;vf=69127290;source=COSMIC) | G/A | NA | A/V | 1218 | COSMIC | Unknown | 0 | RIIIDb |
| [COSM400644](http://www.ensembl.org/Homo_sapiens/Variation/Mappings?db=core;g=ENSG00000113360;r=5:31400604-31532303;t=ENST00000511367;v=COSM400644;vf=69127284;source=COSMIC) | G/A | NA | P/L | 1292 | COSMIC | Unknown | 0 | dsRBD |
| [rs201161679](http://www.ensembl.org/Homo_sapiens/Variation/Mappings?db=core;g=ENSG00000113360;r=5:31400604-31532303;t=ENST00000511367;v=rs201161679;vf=55188382;source=dbSNP) | T/A | NA | Y/F | 1303 | dbSNP | Unknown | 0.05 | dsRBD |
| [COSM592193](http://www.ensembl.org/Homo_sapiens/Variation/Mappings?db=core;g=ENSG00000113360;r=5:31400604-31532303;t=ENST00000511367;v=COSM592193;vf=69127283;source=COSMIC) | C/G | NA | G/R | 1314 | COSMIC | Unknown | 0 | dsRBD |
| [COSM280610](http://www.ensembl.org/Homo_sapiens/Variation/Mappings?db=core;g=ENSG00000113360;r=5:31400604-31532303;t=ENST00000511367;v=COSM280610;vf=69127282;source=COSMIC) | G/A | NA | R/W | 1342 | COSMIC | Unknown | 0 | dsRBD |
| [COSM592194](http://www.ensembl.org/Homo_sapiens/Variation/Mappings?db=core;g=ENSG00000113360;r=5:31400604-31532303;t=ENST00000511367;v=COSM592194;vf=69127281;source=COSMIC) | C/A | NA | R/L | 1342 | COSMIC | Unknown | 0.01 | dsRBD |
| [rs61751196](http://www.ensembl.org/Homo_sapiens/Variation/Mappings?db=core;g=ENSG00000113360;r=5:31400604-31532303;t=ENST00000511367;v=rs61751196;vf=14333332;source=dbSNP) | T/C | NA | R/G | 1364 | dbSNP | Unknown | 0.01 | dsRBD |
| [rs376216811](http://www.ensembl.org/Homo_sapiens/Variation/Mappings?db=core;g=ENSG00000113360;r=5:31400604-31532303;t=ENST00000511367;v=rs376216811;vf=64383813;source=dbSNP) | T/C | NA | D/G | 1367 | dbSNP | ESP | 0.01 | dsRBD |
| **Frameshift** | | | | | | | | |
| [COSM1436994](http://www.ensembl.org/Homo_sapiens/Variation/Mappings?db=core;g=ENSG00000113360;r=5:31400604-31532303;t=ENST00000511367;v=COSM1436994;vf=69127376;source=COSMIC) | G/- (deletion) | NA |  | 157 | COSMIC | Unknown | ND | Pro-rich |
| [COSM1436993](http://www.ensembl.org/Homo_sapiens/Variation/Mappings?db=core;g=ENSG00000113360;r=5:31400604-31532303;t=ENST00000511367;v=COSM1436993;vf=69127359;source=COSMIC) | T/- (deletion) | NA |  | 309 | COSMIC | Unknown | ND | R-rich |
| [COSM272238](http://www.ensembl.org/Homo_sapiens/Variation/Mappings?db=core;g=ENSG00000113360;r=5:31400604-31532303;t=ENST00000511367;v=COSM272238;vf=69127331;source=COSMIC) | -/G (insertion) | NA |  | 612 | COSMIC | Unknown | ND | - |
| [rs35745393](http://www.ensembl.org/Homo_sapiens/Variation/Mappings?db=core;g=ENSG00000113360;r=5:31400604-31532303;t=ENST00000511367;v=rs35745393;vf=11966021;source=dbSNP) | -/C (insertion) | NA |  | 1288 | dbSNP | Unknown | ND | dsRBD |
| [rs145849422](http://www.ensembl.org/Homo_sapiens/Variation/Mappings?db=core;g=ENSG00000113360;r=5:31400604-31532303;t=ENST00000511367;v=rs145849422;vf=36400790;source=dbSNP) | -/C/CC (insertion) | NA |  | 1302 | dbSNP | MO | ND | dsRBD |
| **Stop gained** | | | | | | | | |
| [rs373494822](http://www.ensembl.org/Homo_sapiens/Variation/Mappings?db=core;g=ENSG00000113360;r=5:31400604-31532303;t=ENST00000511367;v=rs373494822;vf=61822760;source=dbSNP) | G/A | NA | R/* | 16 | dbSNP | ESP | ND | Pro-rich |
| [COSM185379](http://www.ensembl.org/Homo_sapiens/Variation/Mappings?db=core;g=ENSG00000113360;r=5:31400604-31532303;t=ENST00000511367;v=COSM185379;vf=69127392;source=COSMIC) | G/A | NA | R/* | 75 | COSMIC | Unknown | ND | Pro-rich |
| [COSM1223870](http://www.ensembl.org/Homo_sapiens/Variation/Mappings?db=core;g=ENSG00000113360;r=5:31400604-31532303;t=ENST00000511367;v=COSM1223870;vf=69127368;source=COSMIC) | G/A | NA | R/* | 271 | COSMIC | Unknown | ND | R-rich |
| [COSM1067239](http://www.ensembl.org/Homo_sapiens/Variation/Mappings?db=core;g=ENSG00000113360;r=5:31400604-31532303;t=ENST00000511367;v=COSM1067239;vf=69127364;source=COSMIC) | G/A | NA | R/* | 287 | COSMIC | Unknown | ND | R-rich |
| [COSM1067237](http://www.ensembl.org/Homo_sapiens/Variation/Mappings?db=core;g=ENSG00000113360;r=5:31400604-31532303;t=ENST00000511367;v=COSM1067237;vf=69127363;source=COSMIC) | G/A | NA | R/* | 295 | COSMIC | Unknown | ND | R-rich |
| [COSM280613](http://www.ensembl.org/Homo_sapiens/Variation/Mappings?db=core;g=ENSG00000113360;r=5:31400604-31532303;t=ENST00000511367;v=COSM280613;vf=69127354;source=COSMIC) | C/A | NA | E/* | 368 | COSMIC | Unknown | ND | - |
| [rs370301677](http://www.ensembl.org/Homo_sapiens/Variation/Mappings?db=core;g=ENSG00000113360;r=5:31400604-31532303;t=ENST00000511367;v=rs370301677;vf=58829652;source=dbSNP) | A/T | NA | L/* | 782 | dbSNP | ESP | ND | - |
| [COSM1543326](http://www.ensembl.org/Homo_sapiens/Variation/Mappings?db=core;g=ENSG00000113360;r=5:31400604-31532303;t=ENST00000511367;v=COSM1543326;vf=69127317;source=COSMIC) | TC/AT | NA | MR/I* | 834 | COSMIC | Unknown | ND | - |
| [COSM482705](http://www.ensembl.org/Homo_sapiens/Variation/Mappings?db=core;g=ENSG00000113360;r=5:31400604-31532303;t=ENST00000511367;v=COSM482705;vf=69127298;source=COSMIC) | C/A | NA | E/* | 1090 | COSMIC | Unknown | ND | - |
| [COSM592192](http://www.ensembl.org/Homo_sapiens/Variation/Mappings?db=core;g=ENSG00000113360;r=5:31400604-31532303;t=ENST00000511367;v=COSM592192;vf=69127291;source=COSMIC) | C/A | NA | E/* | 1193 | COSMIC | Unknown | ND | RIIIDb |
| [COSM738165](http://www.ensembl.org/Homo_sapiens/Variation/Mappings?db=core;g=ENSG00000113360;r=5:31400604-31532303;t=ENST00000511367;v=COSM738165;vf=69127287;source=COSMIC) | C/A | NA | E/* | 1275 | COSMIC | Unknown | ND | dsRBD |

^RefSeq NM_013235.4^

**Legend:** AA = amino acid, ESP = Exome sequencing project, MO = multiple observation, Pro-rich = proline rich, R-rich = arginine rich, RIIID = ribonuclease III domain, dsRBD = double-stranded RNA binding domain, NA = not available, / = not applicable, ND = not determined, - = SNP not residing within domain.
