# Supplementary material for: Genetic variability of microRNA regulome in human
Source: Mol Genet Genomic Med. 2014 Sep 15;3(1):30–9. doi: 10.1002/mgg3.110 (PMC4299713; doi:10.1002/mgg3.110)
Supplement: Table S3 — Catalog of genetic variability residing within DGCR8 gene. [file mgg30003-0030-sd5.docx]

Supplementary table 2: Catalog of genetic variability residing within *DGCR8* gene.

| **Polymorphism ID** | **SNP alleles** | **MAF** | **AA**  **alteration** | **AA coordinates** | **Source** | **Evidence** | **SIFT value** | **Domain** |
| --- | --- | --- | --- | --- | --- | --- | --- | --- |
| **Missense** | | | | | | | | |
| [rs76514473](http://www.ensembl.org/Homo_sapiens/Variation/Mappings?db=core;g=ENSG00000128191;r=22:20067755-20099400;t=ENST00000351989;v=rs76514473;vf=20097694;source=dbSNP) | T/G | NA | V/G | 19 | dbSNP | Unknown | 0.03 | - |
| [COSM1032175](http://www.ensembl.org/Homo_sapiens/Variation/Mappings?db=core;g=ENSG00000128191;r=22:20067755-20099400;t=ENST00000351989;v=COSM1032175;vf=69096040;source=COSMIC) | C/T | NA | T/M | 42 | COSMIC | Unknown | 0 | - |
| [COSM122562](http://www.ensembl.org/Homo_sapiens/Variation/Mappings?db=core;g=ENSG00000128191;r=22:20067755-20099400;t=ENST00000351989;v=COSM122562;vf=69096041;source=COSMIC) | A/G | NA | S/G | 44 | COSMIC | Unknown | 0 | - |
| [COSM160402](http://www.ensembl.org/Homo_sapiens/Variation/Mappings?db=core;g=ENSG00000128191;r=22:20067755-20099400;t=ENST00000351989;v=COSM160402;vf=69096042;source=COSMIC) | G/C | NA | E/Q | 47 | COSMIC | Unknown | 0.02 | - |
| [rs5748529](http://www.ensembl.org/Homo_sapiens/Variation/Mappings?db=core;g=ENSG00000128191;r=22:20067755-20099400;t=ENST00000351989;v=rs5748529;vf=3909594;source=dbSNP) | C/T | NA | A/V | 63 | dbSNP | Unknown | 0.03 | - |
| [rs182736423](http://www.ensembl.org/Homo_sapiens/Variation/Mappings?db=core;g=ENSG00000128191;r=22:20067755-20099400;t=ENST00000351989;v=rs182736423;vf=43016323;source=dbSNP) | C/T | T=0.0005/1 | P/L | 96 | dbSNP | 1000 Genomes | 0 | - |
| [rs150234024](http://www.ensembl.org/Homo_sapiens/Variation/Mappings?db=core;g=ENSG00000128191;r=22:20067755-20099400;t=ENST00000351989;v=rs150234024;vf=40051298;source=dbSNP) | G/A | NA | A/T | 99 | dbSNP | ESP | 0 | - |
| [rs138886652](http://www.ensembl.org/Homo_sapiens/Variation/Mappings?db=core;g=ENSG00000128191;r=22:20067755-20099400;t=ENST00000351989;v=rs138886652;vf=30421807;source=dbSNP) | C/T | NA | P/S | 110 | dbSNP | ESP | 0 | - |
| [rs369938408](http://www.ensembl.org/Homo_sapiens/Variation/Mappings?db=core;g=ENSG00000128191;r=22:20067755-20099400;t=ENST00000351989;v=rs369938408;vf=58483565;source=dbSNP) | G/A | NA | V/M | 122 | dbSNP | ESP | 0 | - |
| [rs118025402](http://www.ensembl.org/Homo_sapiens/Variation/Mappings?db=core;g=ENSG00000128191;r=22:20067755-20099400;t=ENST00000351989;v=rs118025402;vf=29467414;source=dbSNP) | C/T | T=0.0009/2 | R/W | 145 | dbSNP | ESP, 1000 Genomes, MO | 0.02 | - |
| [COSM319871](http://www.ensembl.org/Homo_sapiens/Variation/Mappings?db=core;g=ENSG00000128191;r=22:20067755-20099400;t=ENST00000351989;v=COSM319871;vf=69096053;source=COSMIC) | A/G | NA | Q/R | 226 | COSMIC | Unknown | 0.02 | - |
| [COSM1661436](http://www.ensembl.org/Homo_sapiens/Variation/Mappings?db=core;g=ENSG00000128191;r=22:20067755-20099400;t=ENST00000351989;v=COSM1661436;vf=69096057;source=COSMIC) | A/G | NA | K/E | 289 | COSMIC | Unknown | 0 | - |
| [COSM1250001](http://www.ensembl.org/Homo_sapiens/Variation/Mappings?db=core;g=ENSG00000128191;r=22:20067755-20099400;t=ENST00000351989;v=COSM1250001;vf=69096058;source=COSMIC) | T/C | NA | V/A | 291 | COSMIC | Unknown | 0 | - |
| [COSM182240](http://www.ensembl.org/Homo_sapiens/Variation/Mappings?db=core;g=ENSG00000128191;r=22:20067755-20099400;t=ENST00000351989;v=COSM182240;vf=69096060;source=COSMIC) | G/A | NA | G/E | 336 | COSMIC | Unknown | 0 | - |
| [rs200553015](http://www.ensembl.org/Homo_sapiens/Variation/Mappings?db=core;g=ENSG00000128191;r=22:20067755-20099400;t=ENST00000351989;v=rs200553015;vf=54637694;source=dbSNP) | A/G | G=0.0005/1 | I/V | 350 | dbSNP | MO | 0.05 | - |
| [rs369018600](http://www.ensembl.org/Homo_sapiens/Variation/Mappings?db=core;g=ENSG00000128191;r=22:20067755-20099400;t=ENST00000351989;v=rs369018600;vf=57681970;source=dbSNP) | C/G | NA | R/G | 447 | dbSNP | ESP | 0.03 | - |
| [COSM1414925](http://www.ensembl.org/Homo_sapiens/Variation/Mappings?db=core;g=ENSG00000128191;r=22:20067755-20099400;t=ENST00000351989;v=COSM1414925;vf=69096066;source=COSMIC) | T/C | NA | F/L | 448 | COSMIC | Unknown | 0 | - |
| [COSM1203435](http://www.ensembl.org/Homo_sapiens/Variation/Mappings?db=core;g=ENSG00000128191;r=22:20067755-20099400;t=ENST00000351989;v=COSM1203435;vf=69096067;source=COSMIC) | A/C | NA | K/T | 457 | COSMIC | Unknown | 0.02 | - |
| [COSM1032182](http://www.ensembl.org/Homo_sapiens/Variation/Mappings?db=core;g=ENSG00000128191;r=22:20067755-20099400;t=ENST00000351989;v=COSM1032182;vf=69096069;source=COSMIC) | C/T | NA | R/W | 464 | COSMIC | Unknown | 0 | - |
| [COSM1195273](http://www.ensembl.org/Homo_sapiens/Variation/Mappings?db=core;g=ENSG00000128191;r=22:20067755-20099400;t=ENST00000351989;v=COSM1195273;vf=69096074;source=COSMIC) | G/A | NA | R/H | 527 | COSMIC | Unknown | 0.04 | dsRBD1 |
| [COSM1203436](http://www.ensembl.org/Homo_sapiens/Variation/Mappings?db=core;g=ENSG00000128191;r=22:20067755-20099400;t=ENST00000351989;v=COSM1203436;vf=69096075;source=COSMIC) | C/A | NA | F/L | 532 | COSMIC | Unknown | 0.02 | dsRBD1 |
| [COSM1032184](http://www.ensembl.org/Homo_sapiens/Variation/Mappings?db=core;g=ENSG00000128191;r=22:20067755-20099400;t=ENST00000351989;v=COSM1032184;vf=69096077;source=COSMIC) | T/C | NA | S/P | 555 | COSMIC | Unknown | 0.01 | dsRBD1 |
| [COSM725584](http://www.ensembl.org/Homo_sapiens/Variation/Mappings?db=core;g=ENSG00000128191;r=22:20067755-20099400;t=ENST00000351989;v=COSM725584;vf=69096078;source=COSMIC) | G/T | NA | S/I | 560 | COSMIC | Unknown | 0 | dsRBD1 |
| [rs143256550](http://www.ensembl.org/Homo_sapiens/Variation/Mappings?db=core;g=ENSG00000128191;r=22:20067755-20099400;t=ENST00000351989;v=rs143256550;vf=34149089;source=dbSNP) | G/A | NA | R/H | 663 | dbSNP | ESP | 0.05 | dsRBD2 |
| [rs9606253](http://www.ensembl.org/Homo_sapiens/Variation/Mappings?db=core;g=ENSG00000128191;r=22:20067755-20099400;t=ENST00000351989;v=rs9606253;vf=6172178;source=dbSNP) | G/T | NA | K/N | 713 | dbSNP | HapMap, MO | 0.02 | - |
| [rs11546015](http://www.ensembl.org/Homo_sapiens/Variation/Mappings?db=core;g=ENSG00000128191;r=22:20067755-20099400;t=ENST00000351989;v=rs11546015;vf=7812677;source=dbSNP) | A/G | NA | N/D | 725 | dbSNP | HapMap | 0.03 | - |
| **Stop gained** | | | | | | | | |
| [rs2106143](http://www.ensembl.org/Homo_sapiens/Variation/Mappings?db=core;g=ENSG00000128191;r=22:20067755-20099400;t=ENST00000351989;v=rs2106143;vf=1671796;source=dbSNP) | G/T | NA | E/* | 5 | dbSNP | MO | ND | - |
| [COSM350288](http://www.ensembl.org/Homo_sapiens/Variation/Mappings?db=core;g=ENSG00000128191;r=22:20067755-20099400;t=ENST00000351989;v=COSM350288;vf=69096045;source=COSMIC) | G/T | NA | G/* | 71 | COSMIC | Unknown | ND | - |
| [COSM337709](http://www.ensembl.org/Homo_sapiens/Variation/Mappings?db=core;g=ENSG00000128191;r=22:20067755-20099400;t=ENST00000351989;v=COSM337709;vf=69096055;source=COSMIC) | G/T | NA | E/* | 252 | COSMIC | Unknown | ND | - |
| [COSM1682110](http://www.ensembl.org/Homo_sapiens/Variation/Mappings?db=core;g=ENSG00000128191;r=22:20067755-20099400;t=ENST00000351989;v=COSM1682110;vf=69096065;source=COSMIC) | C/T | NA | R/* | 441 | COSMIC | Unknown | ND | - |
| [COSM1471408](http://www.ensembl.org/Homo_sapiens/Variation/Mappings?db=core;g=ENSG00000128191;r=22:20067755-20099400;t=ENST00000351989;v=COSM1471408;vf=69096079;source=COSMIC) | C/T | NA | R/* | 570 | COSMIC | Unknown |  | dsRBD1 |
| [COSM1535138](http://www.ensembl.org/Homo_sapiens/Variation/Mappings?db=core;g=ENSG00000128191;r=22:20067755-20099400;t=ENST00000351989;v=COSM1535138;vf=69096083;source=COSMIC) | G/T | NA | E/* | 626 | COSMIC | Unknown | ND | dsRBD2 |
| [COSM1032187](http://www.ensembl.org/Homo_sapiens/Variation/Mappings?db=core;g=ENSG00000128191;r=22:20067755-20099400;t=ENST00000351989;v=COSM1032187;vf=69096087;source=COSMIC) | C/T | NA | R/* | 750 | COSMIC | Unknown | ND | - |

^RefSeq NM_022720.6^

**Legend:** AA = amino acid, ESP = Exome sequencing project, MO = multiple observation, dsRBD = double-stranded RNA binding domain, NA = not available, ND = not determined, - = SNP not residing within domain.
