# Supplementary material for: Genetic variability of microRNA regulome in human
Source: Mol Genet Genomic Med. 2014 Sep 15;3(1):30–9. doi: 10.1002/mgg3.110 (PMC4299713; doi:10.1002/mgg3.110)
Supplement: Table S4 — Catalog of genetic variability residing within DICER1 gene. [file mgg30003-0030-sd6.docx]

Supplementary table 3: Catalog of genetic variability residing within *DICER1* gene.

| **Polymorphism ID** | **SNP alleles** | **MAF** | **AA**  **alteration** | **AA coordinates** | **Source** | **Evidence** | **SIFT value** | **Domain** |
| --- | --- | --- | --- | --- | --- | --- | --- | --- |
| **Missense** | | | | | | | | |
| [COSM1608104](http://www.ensembl.org/Homo_sapiens/Variation/Mappings?db=core;g=ENSG00000100697;r=14:95552565-95624347;t=ENST00000526495;v=COSM1608104;vf=69099715;source=COSMIC) | A>T | NA | L>H | 9 | COSMIC | Unknown | 0 | - |
| [rs147660793](http://www.ensembl.org/Homo_sapiens/Variation/Mappings?db=core;g=ENSG00000100697;r=14:95552565-95624347;t=ENST00000526495;v=rs147660793;vf=37840120;source=dbSNP) | G>A | NA | A>V | 20 | dbSNP | ESP, MO | 0.01 | - |
| [COSM470393](http://www.ensembl.org/Homo_sapiens/Variation/Mappings?db=core;g=ENSG00000100697;r=14:95552565-95624347;t=ENST00000526495;v=COSM470393;vf=69099714;source=COSMIC) | G>T | NA | P>Q | 23 | COSMIC | Unknown | 0.01 | - |
| [rs201358110](http://www.ensembl.org/Homo_sapiens/Variation/Mappings?db=core;g=ENSG00000100697;r=14:95552565-95624347;t=ENST00000526495;v=rs201358110;vf=55381515;source=dbSNP) | G>A | NA | P>L | 26 | dbSNP |  | 0.01 | - |
| [rs61729796](http://www.ensembl.org/Homo_sapiens/Variation/Mappings?db=core;g=ENSG00000100697;r=14:95552565-95624347;t=ENST00000526495;v=rs61729796;vf=14327709;source=dbSNP) | A>C | NA | F>C | 28 | dbSNP |  | 0 | - |
| [COSM959285](http://www.ensembl.org/Homo_sapiens/Variation/Mappings?db=core;g=ENSG00000100697;r=14:95552565-95624347;t=ENST00000526495;v=COSM959285;vf=69099713;source=COSMIC) | G>A | NA | P>S | 31 | COSMIC | Unknown | 0.03 | - |
| [COSM959284](http://www.ensembl.org/Homo_sapiens/Variation/Mappings?db=core;g=ENSG00000100697;r=14:95552565-95624347;t=ENST00000526495;v=COSM959284;vf=69099712;source=COSMIC) | A>G | NA | W>R | 32 | COSMIC | Unknown | 0 | - |
| [COSM1629450](http://www.ensembl.org/Homo_sapiens/Variation/Mappings?db=core;g=ENSG00000100697;r=14:95552565-95624347;t=ENST00000526495;v=COSM1629450;vf=69099711;source=COSMIC) | T>G | NA | Q>P | 34 | COSMIC | Unknown | 0.03 | - |
| [rs367797765](http://www.ensembl.org/Homo_sapiens/Variation/Mappings?db=core;g=ENSG00000100697;r=14:95552565-95624347;t=ENST00000526495;v=rs367797765;vf=56580069;source=dbSNP) | G>A | NA | T>M | 43 | dbSNP | ESP | 0 | - |
| [COSM959283](http://www.ensembl.org/Homo_sapiens/Variation/Mappings?db=core;g=ENSG00000100697;r=14:95552565-95624347;t=ENST00000526495;v=COSM959283;vf=69099709;source=COSMIC) | G>A | NA | T>M | 43 | COSMIC | Unknown | 0 | - |
| [rs376657431](http://www.ensembl.org/Homo_sapiens/Variation/Mappings?db=core;g=ENSG00000100697;r=14:95552565-95624347;t=ENST00000526495;v=rs376657431;vf=64824800;source=dbSNP) | G>C | NA | T>R | 71 | dbSNP | ESP | 0.01 | Helicase ATP-binding |
| [COSM1516565](http://www.ensembl.org/Homo_sapiens/Variation/Mappings?db=core;g=ENSG00000100697;r=14:95552565-95624347;t=ENST00000526495;v=COSM1516565;vf=69099705;source=COSMIC) | T>C | NA | R>G | 95 | COSMIC | Unknown | 0.01 | Helicase ATP-binding |
| [COSM1238673](http://www.ensembl.org/Homo_sapiens/Variation/Mappings?db=core;g=ENSG00000100697;r=14:95552565-95624347;t=ENST00000526495;v=COSM1238673;vf=69099701;source=COSMIC) | C>T | NA | R>H | 201 | COSMIC | Unknown | 0.04 | Helicase ATP-binding |
| [COSM959279](http://www.ensembl.org/Homo_sapiens/Variation/Mappings?db=core;g=ENSG00000100697;r=14:95552565-95624347;t=ENST00000526495;v=COSM959279;vf=69099690;source=COSMIC) | T>G | NA | H>P | 341 | COSMIC | Unknown | 0.03 | - |
| [rs148758903](http://www.ensembl.org/Homo_sapiens/Variation/Mappings?db=core;g=ENSG00000100697;r=14:95552565-95624347;t=ENST00000526495;v=rs148758903;vf=38771091;source=dbSNP) | G>C | C=0.0005/1 | P>R | 375 | dbSNP | ESP, 1000 Genomes, MO | 0 | - |
| [COSM1629449](http://www.ensembl.org/Homo_sapiens/Variation/Mappings?db=core;g=ENSG00000100697;r=14:95552565-95624347;t=ENST00000526495;v=COSM1629449;vf=69099686;source=COSMIC) | C>T | NA | E>K | 382 | COSMIC | Unknown | 0.03 | - |
| [COSM1300937](http://www.ensembl.org/Homo_sapiens/Variation/Mappings?db=core;g=ENSG00000100697;r=14:95552565-95624347;t=ENST00000526495;v=COSM1300937;vf=69099685;source=COSMIC) | G>A | NA | R>C | 385 | COSMIC | Unknown | 0.01 | - |
| [rs368535616](http://www.ensembl.org/Homo_sapiens/Variation/Mappings?db=core;g=ENSG00000100697;r=14:95552565-95624347;t=ENST00000526495;v=rs368535616;vf=57223894;source=dbSNP) | C>T | NA | A>T | 454 | dbSNP | ESP | 0 | Helicase C-terminal |
| [rs202029334](http://www.ensembl.org/Homo_sapiens/Variation/Mappings?db=core;g=ENSG00000100697;r=14:95552565-95624347;t=ENST00000526495;v=rs202029334;vf=56031187;source=dbSNP) | T>C | NA | I>M | 461 | dbSNP |  | 0.03 | Helicase C-terminal |
| [rs4566088](http://www.ensembl.org/Homo_sapiens/Variation/Mappings?db=core;g=ENSG00000100697;r=14:95552565-95624347;t=ENST00000526495;v=rs4566088;vf=3472212;source=dbSNP) | C>G | NA | R>T | 499 | dbSNP | MO | 0.01 | Helicase C-terminal |
| [COSM1371856](http://www.ensembl.org/Homo_sapiens/Variation/Mappings?db=core;g=ENSG00000100697;r=14:95552565-95624347;t=ENST00000526495;v=COSM1371856;vf=69099677;source=COSMIC) | T>C | NA | E>G | 502 | COSMIC | Unknown | 0 | Helicase C-terminal |
| [COSM24655](http://www.ensembl.org/Homo_sapiens/Variation/Mappings?db=core;g=ENSG00000100697;r=14:95552565-95624347;t=ENST00000526495;v=COSM24655;vf=69099678;source=COSMIC) | C>T | NA | E>K | 502 | COSMIC | Unknown | 0 | Helicase C-terminal |
| [rs143099538](http://www.ensembl.org/Homo_sapiens/Variation/Mappings?db=core;g=ENSG00000100697;r=14:95552565-95624347;t=ENST00000526495;v=rs143099538;vf=34016332;source=dbSNP) | A>G | NA | I>T | 528 | dbSNP | ESP | 0.01 | Helicase C-terminal |
| [COSM225717](http://www.ensembl.org/Homo_sapiens/Variation/Mappings?db=core;g=ENSG00000100697;r=14:95552565-95624347;t=ENST00000526495;v=COSM225717;vf=69099673;source=COSMIC) | G>A | NA | R>C | 536 | COSMIC | Unknown | 0 | Helicase C-terminal |
| [rs148532788](http://www.ensembl.org/Homo_sapiens/Variation/Mappings?db=core;g=ENSG00000100697;r=14:95552565-95624347;t=ENST00000526495;v=rs148532788;vf=38641947;source=dbSNP) | T>C | C=0.0005/1 | R>G | 554 | dbSNP | 1000 Genomes | 0 | Helicase C-terminal |
| [COSM959273](http://www.ensembl.org/Homo_sapiens/Variation/Mappings?db=core;g=ENSG00000100697;r=14:95552565-95624347;t=ENST00000526495;v=COSM959273;vf=69099669;source=COSMIC) | T>C | NA | Y>C | 560 | COSMIC | Unknown | 0 | Helicase C-terminal |
| [COSM1477878](http://www.ensembl.org/Homo_sapiens/Variation/Mappings?db=core;g=ENSG00000100697;r=14:95552565-95624347;t=ENST00000526495;v=COSM1477878;vf=69099667;source=COSMIC) | G>C | NA | S>C | 591 | COSMIC | Unknown | 0.02 | Helicase C-terminal |
| [rs114947750](http://www.ensembl.org/Homo_sapiens/Variation/Mappings?db=core;g=ENSG00000100697;r=14:95552565-95624347;t=ENST00000526495;v=rs114947750;vf=27562410;source=dbSNP) | C>A | A=0.0009/2 | D>Y | 609 | dbSNP | ESP, 1000 Genomes, MO | 0.03 | - |
| [COSM554120](http://www.ensembl.org/Homo_sapiens/Variation/Mappings?db=core;g=ENSG00000100697;r=14:95552565-95624347;t=ENST00000526495;v=COSM554120;vf=69099665;source=COSMIC) | C>A | NA | D>Y | 620 | COSMIC | Unknown | 0.01 | - |
| [COSM1563024](http://www.ensembl.org/Homo_sapiens/Variation/Mappings?db=core;g=ENSG00000100697;r=14:95552565-95624347;t=ENST00000526495;v=COSM1563024;vf=69099664;source=COSMIC) | G>A | NA | T>M | 629 | COSMIC | Unknown | 0 | - |
| [COSM325152](http://www.ensembl.org/Homo_sapiens/Variation/Mappings?db=core;g=ENSG00000100697;r=14:95552565-95624347;t=ENST00000526495;v=COSM325152;vf=69099663;source=COSMIC) | C>T | NA | D>N | 644 | COSMIC | Unknown | 0 | - |
| [COSM959272](http://www.ensembl.org/Homo_sapiens/Variation/Mappings?db=core;g=ENSG00000100697;r=14:95552565-95624347;t=ENST00000526495;v=COSM959272;vf=69099662;source=COSMIC) | A>G | NA | F>L | 646 | COSMIC | Unknown | 0 | - |
| [rs373914672](http://www.ensembl.org/Homo_sapiens/Variation/Mappings?db=core;g=ENSG00000100697;r=14:95552565-95624347;t=ENST00000526495;v=rs373914672;vf=62259853;source=dbSNP) | G>C | NA | P>R | 683 | dbSNP | ESP | 0.04 | - |
| [rs142300389](http://www.ensembl.org/Homo_sapiens/Variation/Mappings?db=core;g=ENSG00000100697;r=14:95552565-95624347;t=ENST00000526495;v=rs142300389;vf=33320658;source=dbSNP) | T>C | NA | M>V | 684 | dbSNP | ESP | 0.01 | - |
| [COSM699746](http://www.ensembl.org/Homo_sapiens/Variation/Mappings?db=core;g=ENSG00000100697;r=14:95552565-95624347;t=ENST00000526495;v=COSM699746;vf=69099657;source=COSMIC) | C>G | NA | M>I | 684 | COSMIC | Unknown | 0.01 | - |
| [COSM380377](http://www.ensembl.org/Homo_sapiens/Variation/Mappings?db=core;g=ENSG00000100697;r=14:95552565-95624347;t=ENST00000526495;v=COSM380377;vf=69099652;source=COSMIC) | C>A | NA | G>V | 741 | COSMIC | Unknown | 0 | - |
| [COSM1707834](http://www.ensembl.org/Homo_sapiens/Variation/Mappings?db=core;g=ENSG00000100697;r=14:95552565-95624347;t=ENST00000526495;v=COSM1707834;vf=69099649;source=COSMIC) | G>A | NA | P>S | 762 | COSMIC | Unknown | 0.02 | - |
| [COSM433549](http://www.ensembl.org/Homo_sapiens/Variation/Mappings?db=core;g=ENSG00000100697;r=14:95552565-95624347;t=ENST00000526495;v=COSM433549;vf=69099648;source=COSMIC) | C>A | NA | V>F | 776 | COSMIC | Unknown | 0.05 | - |
| [COSM220013](http://www.ensembl.org/Homo_sapiens/Variation/Mappings?db=core;g=ENSG00000100697;r=14:95552565-95624347;t=ENST00000526495;v=COSM220013;vf=69099647;source=COSMIC) | A>G | NA | L>S | 777 | COSMIC | Unknown | 0 | - |
| [COSM699747](http://www.ensembl.org/Homo_sapiens/Variation/Mappings?db=core;g=ENSG00000100697;r=14:95552565-95624347;t=ENST00000526495;v=COSM699747;vf=69099646;source=COSMIC) | G>A | NA | P>L | 780 | COSMIC | Unknown | 0 | - |
| [COSM1371854](http://www.ensembl.org/Homo_sapiens/Variation/Mappings?db=core;g=ENSG00000100697;r=14:95552565-95624347;t=ENST00000526495;v=COSM1371854;vf=69099645;source=COSMIC) | C>T | NA | R>Q | 790 | COSMIC | Unknown | 0.01 | - |
| [COSM74376](http://www.ensembl.org/Homo_sapiens/Variation/Mappings?db=core;g=ENSG00000100697;r=14:95552565-95624347;t=ENST00000526495;v=COSM74376;vf=69099643;source=COSMIC) | G>A | NA | T>M | 806 | COSMIC | Unknown | 0 | - |
| [COSM1371853](http://www.ensembl.org/Homo_sapiens/Variation/Mappings?db=core;g=ENSG00000100697;r=14:95552565-95624347;t=ENST00000526495;v=COSM1371853;vf=69099644;source=COSMIC) | T>C | NA | T>A | 806 | COSMIC | Unknown | 0.01 | - |
| [rs387906934](http://www.ensembl.org/Homo_sapiens/Variation/Mappings?db=core;g=ENSG00000100697;r=14:95552565-95624347;t=ENST00000526495;v=rs387906934;vf=65841939;source=dbSNP) | G>A | NA | S>F | 839 | dbSNP | Unknown | 0.01 | - |
| [COSM959267](http://www.ensembl.org/Homo_sapiens/Variation/Mappings?db=core;g=ENSG00000100697;r=14:95552565-95624347;t=ENST00000526495;v=COSM959267;vf=69099642;source=COSMIC) | G>T | NA | F>L | 854 | COSMIC | Unknown | 0 | - |
| [rs144649926](http://www.ensembl.org/Homo_sapiens/Variation/Mappings?db=core;g=ENSG00000100697;r=14:95552565-95624347;t=ENST00000526495;v=rs144649926;vf=35293957;source=dbSNP) | G>A | NA | R>W | 859 | dbSNP | ESP | 0 | - |
| [rs139441077](http://www.ensembl.org/Homo_sapiens/Variation/Mappings?db=core;g=ENSG00000100697;r=14:95552565-95624347;t=ENST00000526495;v=rs139441077;vf=30888775;source=dbSNP) | G>T | NA | S>Y | 887 | dbSNP | ESP | 0.02 | - |
| [COSM388140](http://www.ensembl.org/Homo_sapiens/Variation/Mappings?db=core;g=ENSG00000100697;r=14:95552565-95624347;t=ENST00000526495;v=COSM388140;vf=69099638;source=COSMIC) | A>T | NA | I>N | 892 | COSMIC | Unknown | 0 | PAZ |
| [COSM554122](http://www.ensembl.org/Homo_sapiens/Variation/Mappings?db=core;g=ENSG00000100697;r=14:95552565-95624347;t=ENST00000526495;v=COSM554122;vf=69099636;source=COSMIC) | C>A | NA | V>F | 921 | COSMIC | Unknown | 0.05 | PAZ |
| [COSM959264](http://www.ensembl.org/Homo_sapiens/Variation/Mappings?db=core;g=ENSG00000100697;r=14:95552565-95624347;t=ENST00000526495;v=COSM959264;vf=69099631;source=COSMIC) | A>C | NA | F>C | 960 | COSMIC | Unknown | 0 | PAZ |
| [COSM959263](http://www.ensembl.org/Homo_sapiens/Variation/Mappings?db=core;g=ENSG00000100697;r=14:95552565-95624347;t=ENST00000526495;v=COSM959263;vf=69099627;source=COSMIC) | G>A | NA | T>I | 993 | COSMIC | Unknown | 0 | PAZ |
| [rs112390607](http://www.ensembl.org/Homo_sapiens/Variation/Mappings?db=core;g=ENSG00000100697;r=14:95552565-95624347;t=ENST00000526495;v=rs112390607;vf=25316419;source=dbSNP) | T>C | NA | T>A | 993 | dbSNP | Unknown | 0.01 | PAZ |
| [COSM1608102](http://www.ensembl.org/Homo_sapiens/Variation/Mappings?db=core;g=ENSG00000100697;r=14:95552565-95624347;t=ENST00000526495;v=COSM1608102;vf=69099626;source=COSMIC) | T>C | NA | I>V | 1032 | COSMIC | Unknown | 0.02 | PAZ |
| [COSM1608101](http://www.ensembl.org/Homo_sapiens/Variation/Mappings?db=core;g=ENSG00000100697;r=14:95552565-95624347;t=ENST00000526495;v=COSM1608101;vf=69099625;source=COSMIC) | T>G | NA | E>A | 1036 | COSMIC | Unknown | 0 | PAZ |
| [COSM1371850](http://www.ensembl.org/Homo_sapiens/Variation/Mappings?db=core;g=ENSG00000100697;r=14:95552565-95624347;t=ENST00000526495;v=COSM1371850;vf=69099623;source=COSMIC) | G>A | NA | L>F | 1054 | COSMIC | Unknown | 0 | - |
| [COSM1203588](http://www.ensembl.org/Homo_sapiens/Variation/Mappings?db=core;g=ENSG00000100697;r=14:95552565-95624347;t=ENST00000526495;v=COSM1203588;vf=69099622;source=COSMIC) | C>T | NA | R>H | 1060 | COSMIC | Unknown | 0 | - |
| [COSM554124](http://www.ensembl.org/Homo_sapiens/Variation/Mappings?db=core;g=ENSG00000100697;r=14:95552565-95624347;t=ENST00000526495;v=COSM554124;vf=69099621;source=COSMIC) | C>T | NA | E>K | 1068 | COSMIC | Unknown | 0.03 | - |
| [COSM302053](http://www.ensembl.org/Homo_sapiens/Variation/Mappings?db=core;g=ENSG00000100697;r=14:95552565-95624347;t=ENST00000526495;v=COSM302053;vf=69099619;source=COSMIC) | T>C | NA | D>G | 1147 | COSMIC | Unknown | 0.02 | - |
| [rs201523588](http://www.ensembl.org/Homo_sapiens/Variation/Mappings?db=core;g=ENSG00000100697;r=14:95552565-95624347;t=ENST00000526495;v=rs201523588;vf=55562217;source=dbSNP) | T>A | A=0.0009/2 | N>I | 1186 | dbSNP | Unknown | 0 | - |
| [COSM699750](http://www.ensembl.org/Homo_sapiens/Variation/Mappings?db=core;g=ENSG00000100697;r=14:95552565-95624347;t=ENST00000526495;v=COSM699750;vf=69099614;source=COSMIC) | C>A | NA | C>F | 1235 | COSMIC | Unknown | 0 | - |
| [rs139346443](http://www.ensembl.org/Homo_sapiens/Variation/Mappings?db=core;g=ENSG00000100697;r=14:95552565-95624347;t=ENST00000526495;v=rs139346443;vf=30894641;source=dbSNP) | G>A | A=0.000/0 | T>M | 1264 | dbSNP | ESP, MO | 0.04 | - |
| [COSM198651](http://www.ensembl.org/Homo_sapiens/Variation/Mappings?db=core;g=ENSG00000100697;r=14:95552565-95624347;t=ENST00000526495;v=COSM198651;vf=69099611;source=COSMIC) | A>T | NA | L>M | 1301 | COSMIC | Unknown | 0.01 | RIIIDa |
| [COSM1666226](http://www.ensembl.org/Homo_sapiens/Variation/Mappings?db=core;g=ENSG00000100697;r=14:95552565-95624347;t=ENST00000526495;v=COSM1666226;vf=69099609;source=COSMIC) | G>C | NA | L>V | 1331 | COSMIC | Unknown | 0 | RIIIDa |
| [COSM959258](http://www.ensembl.org/Homo_sapiens/Variation/Mappings?db=core;g=ENSG00000100697;r=14:95552565-95624347;t=ENST00000526495;v=COSM959258;vf=69099608;source=COSMIC) | G>A | NA | S>L | 1344 | COSMIC | Unknown | 0 | RIIIDa |
| [COSM175437](http://www.ensembl.org/Homo_sapiens/Variation/Mappings?db=core;g=ENSG00000100697;r=14:95552565-95624347;t=ENST00000526495;v=COSM175437;vf=69099606;source=COSMIC) | C>T | NA | R>H | 1358 | COSMIC | Unknown | 0.01 | RIIIDa |
| [COSM959257](http://www.ensembl.org/Homo_sapiens/Variation/Mappings?db=core;g=ENSG00000100697;r=14:95552565-95624347;t=ENST00000526495;v=COSM959257;vf=69099603;source=COSMIC) | T>C | NA | E>G | 1424 | COSMIC | Unknown | 0.04 | - |
| [rs200890670](http://www.ensembl.org/Homo_sapiens/Variation/Mappings?db=core;g=ENSG00000100697;r=14:95552565-95624347;t=ENST00000526495;v=rs200890670;vf=54935203;source=dbSNP) | A>G | G=0.0005/1 | L>P | 1469 | dbSNP | Unknown | 0 | - |
| [COSM959255](http://www.ensembl.org/Homo_sapiens/Variation/Mappings?db=core;g=ENSG00000100697;r=14:95552565-95624347;t=ENST00000526495;v=COSM959255;vf=69099597;source=COSMIC) | T>G | NA | K>N | 1482 | COSMIC | Unknown | 0.01 | - |
| [COSM1516569](http://www.ensembl.org/Homo_sapiens/Variation/Mappings?db=core;g=ENSG00000100697;r=14:95552565-95624347;t=ENST00000526495;v=COSM1516569;vf=69099594;source=COSMIC) | G>A | NA | S>F | 1505 | COSMIC | Unknown | 0.01 | - |
| [COSM1300936](http://www.ensembl.org/Homo_sapiens/Variation/Mappings?db=core;g=ENSG00000100697;r=14:95552565-95624347;t=ENST00000526495;v=COSM1300936;vf=69099593;source=COSMIC) | G>C | NA | S>C | 1505 | COSMIC | Unknown | 0.01 | - |
| [COSM1477876](http://www.ensembl.org/Homo_sapiens/Variation/Mappings?db=core;g=ENSG00000100697;r=14:95552565-95624347;t=ENST00000526495;v=COSM1477876;vf=69099592;source=COSMIC) | C>T | NA | C>Y | 1510 | COSMIC | Unknown | 0 | - |
| [COSM699752](http://www.ensembl.org/Homo_sapiens/Variation/Mappings?db=core;g=ENSG00000100697;r=14:95552565-95624347;t=ENST00000526495;v=COSM699752;vf=69099591;source=COSMIC) | T>A | NA | K>N | 1516 | COSMIC | Unknown | 0 | - |
| [rs113234676](http://www.ensembl.org/Homo_sapiens/Variation/Mappings?db=core;g=ENSG00000100697;r=14:95552565-95624347;t=ENST00000526495;v=rs113234676;vf=26013298;source=dbSNP) | T>C | NA | D>G | 1522 | dbSNP | Unknown | 0.05 | - |
| [COSM1516570](http://www.ensembl.org/Homo_sapiens/Variation/Mappings?db=core;g=ENSG00000100697;r=14:95552565-95624347;t=ENST00000526495;v=COSM1516570;vf=69099588;source=COSMIC) | T>C | NA | K>E | 1557 | COSMIC | Unknown | 0 | - |
| [rs369465519](http://www.ensembl.org/Homo_sapiens/Variation/Mappings?db=core;g=ENSG00000100697;r=14:95552565-95624347;t=ENST00000526495;v=rs369465519;vf=58089330;source=dbSNP) | C>A | NA | Q>H | 1580 | dbSNP | ESP | 0.01 | - |
| [rs137852976](http://www.ensembl.org/Homo_sapiens/Variation/Mappings?db=core;g=ENSG00000100697;r=14:95552565-95624347;t=ENST00000526495;v=rs137852976;vf=29550331;source=dbSNP) | A>C | NA | L>R | 1583 | dbSNP | Unknown | 0 | - |
| [COSM1371847](http://www.ensembl.org/Homo_sapiens/Variation/Mappings?db=core;g=ENSG00000100697;r=14:95552565-95624347;t=ENST00000526495;v=COSM1371847;vf=69099587;source=COSMIC) | G>A | NA | P>L | 1592 | COSMIC | Unknown | 0.01 | - |
| [rs189119295](http://www.ensembl.org/Homo_sapiens/Variation/Mappings?db=core;g=ENSG00000100697;r=14:95552565-95624347;t=ENST00000526495;v=rs189119295;vf=49432444;source=dbSNP) | G>A | A=0.0005/1 | R>W | 1607 | dbSNP | ESP, 1000 Genomes, MO | 0.02 | - |
| [rs146715213](http://www.ensembl.org/Homo_sapiens/Variation/Mappings?db=core;g=ENSG00000100697;r=14:95552565-95624347;t=ENST00000526495;v=rs146715213;vf=37048001;source=dbSNP) | T>C | C=0.0005/1 | M>V | 1649 | dbSNP | 1000 Genomes | 0.03 | - |
| [COSM959252](http://www.ensembl.org/Homo_sapiens/Variation/Mappings?db=core;g=ENSG00000100697;r=14:95552565-95624347;t=ENST00000526495;v=COSM959252;vf=69099583;source=COSMIC) | A>C | NA | F>C | 1650 | COSMIC | Unknown | 0.01 | - |
| [COSM244035](http://www.ensembl.org/Homo_sapiens/Variation/Mappings?db=core;g=ENSG00000100697;r=14:95552565-95624347;t=ENST00000526495;v=COSM244035;vf=69099579;source=COSMIC) | G>A | NA | R>C | 1703 | COSMIC | Unknown | 0 | RIIIDb |
| [COSM959250](http://www.ensembl.org/Homo_sapiens/Variation/Mappings?db=core;g=ENSG00000100697;r=14:95552565-95624347;t=ENST00000526495;v=COSM959250;vf=69099576;source=COSMIC) | T>G | NA | E>A | 1705 | COSMIC | Unknown | 0 | RIIIDb |
| [COSM959251](http://www.ensembl.org/Homo_sapiens/Variation/Mappings?db=core;g=ENSG00000100697;r=14:95552565-95624347;t=ENST00000526495;v=COSM959251;vf=69099578;source=COSMIC) | C>T | NA | E>K | 1705 | COSMIC | Unknown | 0 | RIIIDb |
| [COSM1371844](http://www.ensembl.org/Homo_sapiens/Variation/Mappings?db=core;g=ENSG00000100697;r=14:95552565-95624347;t=ENST00000526495;v=COSM1371844;vf=69099577;source=COSMIC) | C>G | NA | E>Q | 1705 | COSMIC | Unknown | 0 | RIIIDb |
| [COSM959249](http://www.ensembl.org/Homo_sapiens/Variation/Mappings?db=core;g=ENSG00000100697;r=14:95552565-95624347;t=ENST00000526495;v=COSM959249;vf=69099575;source=COSMIC) | C>T | NA | D>N | 1709 | COSMIC | Unknown | 0 | RIIIDb |
| [COSM304354](http://www.ensembl.org/Homo_sapiens/Variation/Mappings?db=core;g=ENSG00000100697;r=14:95552565-95624347;t=ENST00000526495;v=COSM304354;vf=69099574;source=COSMIC) | G>A | NA | P>L | 1729 | COSMIC | Unknown | 0 | RIIIDb |
| [COSM310568](http://www.ensembl.org/Homo_sapiens/Variation/Mappings?db=core;g=ENSG00000100697;r=14:95552565-95624347;t=ENST00000526495;v=COSM310568;vf=69099573;source=COSMIC) | T>C | NA | N>D | 1741 | COSMIC | Unknown | 0 | RIIIDb |
| [COSM1300934](http://www.ensembl.org/Homo_sapiens/Variation/Mappings?db=core;g=ENSG00000100697;r=14:95552565-95624347;t=ENST00000526495;v=COSM1300934;vf=69099572;source=COSMIC) | T>C | NA | N>S | 1742 | COSMIC | Unknown | 0 | RIIIDb |
| [COSM213707](http://www.ensembl.org/Homo_sapiens/Variation/Mappings?db=core;g=ENSG00000100697;r=14:95552565-95624347;t=ENST00000526495;v=COSM213707;vf=69099570;source=COSMIC) | G>A | NA | S>L | 1747 | COSMIC | Unknown | 0.01 | RIIIDb |
| [COSM699753](http://www.ensembl.org/Homo_sapiens/Variation/Mappings?db=core;g=ENSG00000100697;r=14:95552565-95624347;t=ENST00000526495;v=COSM699753;vf=69099569;source=COSMIC) | G>T | NA | H>N | 1755 | COSMIC | Unknown | 0 | RIIIDb |
| [COSM120867](http://www.ensembl.org/Homo_sapiens/Variation/Mappings?db=core;g=ENSG00000100697;r=14:95552565-95624347;t=ENST00000526495;v=COSM120867;vf=69099568;source=COSMIC) | A>C | NA | F>V | 1758 | COSMIC | Unknown | 0.01 | RIIIDb |
| [COSM959247](http://www.ensembl.org/Homo_sapiens/Variation/Mappings?db=core;g=ENSG00000100697;r=14:95552565-95624347;t=ENST00000526495;v=COSM959247;vf=69099566;source=COSMIC) | C>T | NA | E>K | 1803 | COSMIC | Unknown | 0 | RIIIDb |
| [COSM959246](http://www.ensembl.org/Homo_sapiens/Variation/Mappings?db=core;g=ENSG00000100697;r=14:95552565-95624347;t=ENST00000526495;v=COSM959246;vf=69099564;source=COSMIC) | T>G | NA | D>A | 1810 | COSMIC | Unknown | 0 | RIIIDb |
| [COSM1371843](http://www.ensembl.org/Homo_sapiens/Variation/Mappings?db=core;g=ENSG00000100697;r=14:95552565-95624347;t=ENST00000526495;v=COSM1371843;vf=69099565;source=COSMIC) | C>T | NA | D>N | 1810 | COSMIC | Unknown | 0 | RIIIDb |
| [COSM959244](http://www.ensembl.org/Homo_sapiens/Variation/Mappings?db=core;g=ENSG00000100697;r=14:95552565-95624347;t=ENST00000526495;v=COSM959244;vf=69099562;source=COSMIC) | T>C | NA | E>G | 1813 | COSMIC | Unknown | 0 | RIIIDb |
| [COSM959245](http://www.ensembl.org/Homo_sapiens/Variation/Mappings?db=core;g=ENSG00000100697;r=14:95552565-95624347;t=ENST00000526495;v=COSM959245;vf=69099561;source=COSMIC) | T>G | NA | E>A | 1813 | COSMIC | Unknown | 0 | RIIIDb |
| [COSM1203589](http://www.ensembl.org/Homo_sapiens/Variation/Mappings?db=core;g=ENSG00000100697;r=14:95552565-95624347;t=ENST00000526495;v=COSM1203589;vf=69099563;source=COSMIC) | C>G | NA | E>Q | 1813 | COSMIC | Unknown | 0 | RIIIDb |
| [COSM959243](http://www.ensembl.org/Homo_sapiens/Variation/Mappings?db=core;g=ENSG00000100697;r=14:95552565-95624347;t=ENST00000526495;v=COSM959243;vf=69099559;source=COSMIC) | C>T | NA | M>I | 1821 | COSMIC | Unknown | 0.02 | RIIIDb |
| [rs377685186](http://www.ensembl.org/Homo_sapiens/Variation/Mappings?db=core;g=ENSG00000100697;r=14:95552565-95624347;t=ENST00000526495;v=rs377685186;vf=65796643;source=dbSNP) | C>A | NA | G>V | 1824 | dbSNP | ESP | 0 | RIIIDb |
| [COSM1678225](http://www.ensembl.org/Homo_sapiens/Variation/Mappings?db=core;g=ENSG00000100697;r=14:95552565-95624347;t=ENST00000526495;v=COSM1678225;vf=69099558;source=COSMIC) | C>T | NA | M>I | 1825 | COSMIC | Unknown | 0.01 | - |
| [COSM699755](http://www.ensembl.org/Homo_sapiens/Variation/Mappings?db=core;g=ENSG00000100697;r=14:95552565-95624347;t=ENST00000526495;v=COSM699755;vf=69099555;source=COSMIC) | G>A | NA | R>C | 1851 | COSMIC | Unknown | 0 | dsRBD |
| [COSM198650](http://www.ensembl.org/Homo_sapiens/Variation/Mappings?db=core;g=ENSG00000100697;r=14:95552565-95624347;t=ENST00000526495;v=COSM198650;vf=69099552;source=COSMIC) | C>A | NA | V>F | 1893 | COSMIC | Unknown | 0.01 | dsRBD |
| [COSM959242](http://www.ensembl.org/Homo_sapiens/Variation/Mappings?db=core;g=ENSG00000100697;r=14:95552565-95624347;t=ENST00000526495;v=COSM959242;vf=69099551;source=COSMIC) | G>A | NA | A>V | 1914 | COSMIC | Unknown | 0.03 | dsRBD |
| **Frameshift** | | | | | | | | |
| [COSM959274](http://www.ensembl.org/Homo_sapiens/Variation/Mappings?db=core;g=ENSG00000100697;r=14:95552565-95624347;t=ENST00000526495;v=COSM959274;vf=69099672;source=COSMIC) | A>- | NA | / | 539 | COSMIC | Unknown | ND | Helicase C-terminal |
| [rs34141151](http://www.ensembl.org/Homo_sapiens/Variation/Mappings?db=core;g=ENSG00000100697;r=14:95552565-95624347;t=ENST00000526495;v=rs34141151;vf=10559389;source=dbSNP) | ->A | NA | / | 1164 | dbSNP | Unknown | ND | - |
| [rs35252236](http://www.ensembl.org/Homo_sapiens/Variation/Mappings?db=core;g=ENSG00000100697;r=14:95552565-95624347;t=ENST00000526495;v=rs35252236;vf=11557622;source=dbSNP) | A>- | NA | / | 1323 | dbSNP | Unknown | ND | RIIIDa |
| [rs34678453](http://www.ensembl.org/Homo_sapiens/Variation/Mappings?db=core;g=ENSG00000100697;r=14:95552565-95624347;t=ENST00000526495;v=rs34678453;vf=11053641;source=dbSNP) | ->G | NA | / | 1377 | dbSNP | Unknown | ND | RIIIDa |
| [COSM1371849](http://www.ensembl.org/Homo_sapiens/Variation/Mappings?db=core;g=ENSG00000100697;r=14:95552565-95624347;t=ENST00000526495;v=COSM1371849;vf=69099598;source=COSMIC) | ->A | NA | / | 1473 | COSMIC | Unknown | ND | - |
| [COSM1371848](http://www.ensembl.org/Homo_sapiens/Variation/Mappings?db=core;g=ENSG00000100697;r=14:95552565-95624347;t=ENST00000526495;v=COSM1371848;vf=69099596;source=COSMIC) | T>- | NA | / | 1486 | COSMIC | Unknown | ND | - |
| [COSM1684395](http://www.ensembl.org/Homo_sapiens/Variation/Mappings?db=core;g=ENSG00000100697;r=14:95552565-95624347;t=ENST00000526495;v=COSM1684395;vf=69099590;source=COSMIC) | CA>- | NA | / | 1535 | COSMIC | Unknown | ND | - |
| [COSM236028](http://www.ensembl.org/Homo_sapiens/Variation/Mappings?db=core;g=ENSG00000100697;r=14:95552565-95624347;t=ENST00000526495;v=COSM236028;vf=69099585;source=COSMIC) | CGAGTAGGGC>- | NA | / | 1604 | COSMIC | Unknown | ND | - |
| **Stop gained** | | | | | | | | |
| [COSM297364](http://www.ensembl.org/Homo_sapiens/Variation/Mappings?db=core;g=ENSG00000100697;r=14:95552565-95624347;t=ENST00000526495;v=COSM297364;vf=69099700;source=COSMIC) | C>A | NA | E>* | 218 | COSMIC | Unknown | ND | Helicase ATP-binding |
| [rs12432511](http://www.ensembl.org/Homo_sapiens/Variation/Mappings?db=core;g=ENSG00000100697;r=14:95552565-95624347;t=ENST00000526495;v=rs12432511;vf=8682999;source=dbSNP) | A>T | NA | C>* | 257 | dbSNP | Unknown | ND | - |
| [COSM1371857](http://www.ensembl.org/Homo_sapiens/Variation/Mappings?db=core;g=ENSG00000100697;r=14:95552565-95624347;t=ENST00000526495;v=COSM1371857;vf=69099691;source=COSMIC) | C>A | NA | E>* | 328 | COSMIC | Unknown | ND | - |
| [COSM198667](http://www.ensembl.org/Homo_sapiens/Variation/Mappings?db=core;g=ENSG00000100697;r=14:95552565-95624347;t=ENST00000526495;v=COSM198667;vf=69099683;source=COSMIC) | G>A | NA | Q>* | 406 | COSMIC | Unknown | ND | - |
| [COSM959276](http://www.ensembl.org/Homo_sapiens/Variation/Mappings?db=core;g=ENSG00000100697;r=14:95552565-95624347;t=ENST00000526495;v=COSM959276;vf=69099682;source=COSMIC) | C>A | NA | E>* | 426 | COSMIC | Unknown | ND | - |
| [rs137852977](http://www.ensembl.org/Homo_sapiens/Variation/Mappings?db=core;g=ENSG00000100697;r=14:95552565-95624347;t=ENST00000526495;v=rs137852977;vf=29550332;source=dbSNP) | C>A | NA | E>* | 503 | dbSNP | Unknown | ND | Helicase C-terminal |
| [COSM198659](http://www.ensembl.org/Homo_sapiens/Variation/Mappings?db=core;g=ENSG00000100697;r=14:95552565-95624347;t=ENST00000526495;v=COSM198659;vf=69099674;source=COSMIC) | C>A | NA | E>* | 524 | COSMIC | Unknown | ND | Helicase C-terminal |
| [rs137852979](http://www.ensembl.org/Homo_sapiens/Variation/Mappings?db=core;g=ENSG00000100697;r=14:95552565-95624347;t=ENST00000526495;v=rs137852979;vf=29550334;source=dbSNP) | G>A | NA | R>* | 544 | dbSNP | Unknown | ND | Helicase C-terminal |
| [COSM393242](http://www.ensembl.org/Homo_sapiens/Variation/Mappings?db=core;g=ENSG00000100697;r=14:95552565-95624347;t=ENST00000526495;v=COSM393242;vf=69099656;source=COSMIC) | G>A | NA | R>* | 688 | COSMIC | Unknown | ND | - |
| [rs137852978](http://www.ensembl.org/Homo_sapiens/Variation/Mappings?db=core;g=ENSG00000100697;r=14:95552565-95624347;t=ENST00000526495;v=rs137852978;vf=29550333;source=dbSNP) | G>A | NA | R>* | 944 | dbSNP | Unknown | ND | PAZ |
| [COSM1579425](http://www.ensembl.org/Homo_sapiens/Variation/Mappings?db=core;g=ENSG00000100697;r=14:95552565-95624347;t=ENST00000526495;v=COSM1579425;vf=69099612;source=COSMIC) | G>A | NA | Q>* | 1299 | COSMIC | Unknown | ND | RIIIDa |

^RefSeq NM_177438.2^

**Legend:** AA = amino acid, ESP = Exome sequencing project, MO = multiple observation, RIIID = ribonuclease III domain, dsRBD = double-stranded RNA binding domain, NA = not available, / = not applicable, ND = not determined, - = SNP not residing within domain.
