# Supplementary material for: Genetic variability of microRNA regulome in human
Source: Mol Genet Genomic Med. 2014 Sep 15;3(1):30–9. doi: 10.1002/mgg3.110 (PMC4299713; doi:10.1002/mgg3.110)
Supplement: Table S5 — Catalog of genetic variability residing within Drosha and Dicer cleavage sites. [file mgg30003-0030-sd7.docx]

Supplementary Table 4: Catalog of genetic variability residing within Drosha and Dicer cleavage sites.

| **micro RNA** | **Strand** | **A** | **B** | **C** | **D** | **Polymorphism ID** | **Type of polymorphism** | **Validation status** | **MAF** | **Experiment** |
| --- | --- | --- | --- | --- | --- | --- | --- | --- | --- | --- |
| hsa-mir-16-1 / *DLEU2* | 3p |  |  |  | x | [rs72631826](http://www.ncbi.nlm.nih.gov/projects/SNP/snp_ref.cgi?rs=rs72631826) | A>G | Validated | G=0.007/16 |  |
| hsa-mir-26a-1 / *CTDSPL* | 3p |  | x |  |  | [rs182070256](http://www.ncbi.nlm.nih.gov/projects/SNP/snp_ref.cgi?rs=rs182070256) | G>A | Validated | A=0.001/2 |  |
| hsa-mir-10a / *HOXB3, HOXB4* | 5p | x |  |  |  | [rs72631828](http://www.ncbi.nlm.nih.gov/projects/SNP/snp_ref.cgi?rs=rs72631828) | T>C | Validated | NA |  |
| hsa-mir-27b / *C9orf3* | 3p |  | x |  |  | [rs192552111](http://www.ncbi.nlm.nih.gov/projects/SNP/snp_ref.cgi?rs=rs192552111) | C>T | Validated | T=0.000/1 |  |
| hsa-mir-140 / *WWP2* | 5p | x |  |  |  | [rs7205289](http://www.ncbi.nlm.nih.gov/projects/SNP/snp_ref.cgi?rs=rs7205289) | C>A | Unknown | NA |  |
| hsa-mir-18a / *MIR17HG* | 5p | x |  |  |  | [rs41275866](http://www.ncbi.nlm.nih.gov/projects/SNP/snp_ref.cgi?rs=rs41275866) | C>G | Unknown | NA |  |
| hsa-mir-154 / intergenic | 5p |  |  | x |  | [rs41286570](http://www.ncbi.nlm.nih.gov/projects/SNP/snp_ref.cgi?rs=rs41286570) | G>A | Validated | A=0.001/2 |  |
| hsa-mir-183 / intergenic | 5p |  |  |  | x | [rs41281222](http://www.ncbi.nlm.nih.gov/projects/SNP/snp_ref.cgi?rs=rs41281222) | C>T | Validated | NA |  |
| hsa-mir-339 / *C7orf50* | 5p |  |  | x |  | rs145196722 | C>T | Validated | T=0.001/3 |  |
| hsa-mir-365b / *AC003101.1* | 3p | x |  |  |  | [rs35143473](http://www.ncbi.nlm.nih.gov/projects/SNP/snp_ref.cgi?rs=rs35143473) | T>- | Unknown | NA |  |
| hsa-mir-383 / *SGCZ* | 5p |  | x |  |  | [rs112302475](http://www.ncbi.nlm.nih.gov/projects/SNP/snp_ref.cgi?rs=rs112302475) | G>T | Unknown | NA |  |
| hsa-mir-449c / *CDC20B* | 5p | x |  |  |  | [rs75661995](http://www.ncbi.nlm.nih.gov/projects/SNP/snp_ref.cgi?rs=rs75661995) | C>T | Validated | T=0.001/2 |  |
| hsa-mir-485 / intergenic | 5p |  | x |  |  | [rs112982830](http://www.ncbi.nlm.nih.gov/projects/SNP/snp_ref.cgi?rs=rs112982830) | A>G | Unknown | NA |  |
| hsa-mir-486 / *ANK1* | 3p | x  DNP |  |  |  | [rs59908561](http://www.ncbi.nlm.nih.gov/projects/SNP/snp_ref.cgi?rs=rs59908561) | ->G | Validated | =0.000/0 |  |
|  |  |  |  |  |  | [rs59908561](http://www.ncbi.nlm.nih.gov/projects/SNP/snp_ref.cgi?rs=rs59908561)* | ->G | Validated |  |  |
| hsa-mir-488 / *ASTN1* | 5p |  |  | x |  | [rs181176070](http://www.ncbi.nlm.nih.gov/projects/SNP/snp_ref.cgi?rs=rs181176070) | C>T | Validated | C=0.001/3 |  |
| hsa-mir-518d / intergenic | 3p |  |  |  | x | [rs116220629](http://www.ncbi.nlm.nih.gov/projects/SNP/snp_ref.cgi?rs=rs116220629) | C>T | Validated | T=0.016/34 |  |
| hsa-mir-518d / intergenic | 3p |  | x |  |  | [rs151129856](http://www.ncbi.nlm.nih.gov/projects/SNP/snp_ref.cgi?rs=rs151129856) | C>T | Validated | T=0.000/1 |  |
| hsa-mir-520h / intergenic | 3p |  | x |  |  | [rs111745142](http://www.ncbi.nlm.nih.gov/projects/SNP/snp_ref.cgi?rs=rs111745142) | ->GT | Validated | G=0.022/47 |  |
| hsa-mir-527 / intergenic | 5p |  |  | x |  | [rs186554893](http://www.ncbi.nlm.nih.gov/projects/SNP/snp_ref.cgi?rs=rs186554893) | C>T | Validated | T=0.000/1 |  |
| hsa-mir-532 / *CLCN5* | 3p | x |  |  |  | [rs456615](http://www.ncbi.nlm.nih.gov/projects/SNP/snp_ref.cgi?rs=rs456615) | A>G | Validated | A=0.063/104 |  |
| hsa-mir-548ad / intergenic | 3p |  |  |  | x  DNP | [rs147085950](http://www.ncbi.nlm.nih.gov/projects/SNP/snp_ref.cgi?rs=rs147085950) | C>T | Validated | T=0.001/2 |  |
|  |  |  |  | x  DNP |  | [rs62143301](http://www.ncbi.nlm.nih.gov/projects/SNP/snp_ref.cgi?rs=rs62143301) | G>A | Validated | A=0.049/107 |  |
| hsa-mir-548aj-2 / *SYTL5, TM4SF2* | 3p |  | x |  |  | [rs113330267](http://www.ncbi.nlm.nih.gov/projects/SNP/snp_ref.cgi?rs=rs113330267) | C>T | Validated | NA |  |
| hsa-mir-548am / *CTPS2* | 5p |  | x |  |  | [rs181147953](http://www.ncbi.nlm.nih.gov/projects/SNP/snp_ref.cgi?rs=rs181147953) | T>G | Validated | G=0.001/1 |  |
| hsa-mir-548ao / *SFRP1* | 5p |  |  |  | x | [rs138586956](http://www.ncbi.nlm.nih.gov/projects/SNP/snp_ref.cgi?rs=rs138586956) | C>T | Validated | T=0.001/3 |  |
| hsa-mir-548ao / *SFRP1* | 3p |  |  | x |  | [rs13272265](http://www.ncbi.nlm.nih.gov/projects/SNP/snp_ref.cgi?rs=rs13272265) | C>T | Validated | NA |  |
| hsa-mir-548ap / intergenic | 5p |  |  |  | x | [rs76468441](http://www.ncbi.nlm.nih.gov/projects/SNP/snp_ref.cgi?rs=rs76468441) | C>T | Validated | T=0.031/68 |  |
| hsa-mir-548b / *FAM184A* | 5p |  |  | x |  | [rs188182113](http://www.ncbi.nlm.nih.gov/projects/SNP/snp_ref.cgi?rs=rs188182113) | G>C | Validated | T=0.031/68 |  |
| hsa-mir-548l / *MRE11A* | 5p |  |  | x  DNP |  | [rs148377174](http://www.ncbi.nlm.nih.gov/projects/SNP/snp_ref.cgi?rs=rs148377174) | G>A | Validated | A=0.000/1 |  |
|  |  |  |  |  | x  DNP | [rs11020790](http://www.ncbi.nlm.nih.gov/projects/SNP/snp_ref.cgi?rs=rs11020790) | C>T | Validated | T=0.041/89 |  |
| hsa-mir-548o / *PRKRIP1* | 3p |  |  |  | x | [rs181862774](http://www.ncbi.nlm.nih.gov/projects/SNP/snp_ref.cgi?rs=rs181862774) | A>G | Validated | G=0.001/3 |  |
| hsa-mir-550a-2 / *AVL9; AC018633.4* - antisense | 5p |  |  |  | x | [rs113464681](http://www.ncbi.nlm.nih.gov/projects/SNP/snp_ref.cgi?rs=rs113464681) | TGT>- | Unknown | NA |  |
| hsa-mir-550a-3 / *AC007255.7; AC007255.*5 - antisense | 5p |  |  |  | x | [rs850108](http://www.ncbi.nlm.nih.gov/projects/SNP/snp_ref.cgi?rs=rs850108) | A>G | Validated | G=0.178/388 |  |
| hsa-mir-559 / *EPCAM* | 5p |  | x |  |  | [rs138461304](http://www.ncbi.nlm.nih.gov/projects/SNP/snp_ref.cgi?rs=rs138461304) | TAAAG>- | Validated | NA | Bhattacharya et al., 2012 |
| hsa-mir-562 / *DIS3L2* | 3p | x | x |  |  | rs140596642 | CTGTACCATTTGCACTCC>- | Validated | NA | Bhattacharya et al., 2012; Drake et al., 2009 |
| hsa-mir-581 / *ARL15* | 5p |  |  | x |  | [rs1694089](http://www.ncbi.nlm.nih.gov/projects/SNP/snp_ref.cgi?rs=rs1694089) | G>T | Validated | NA |  |
| hsa-mir-607 / intergenic | 3p | x |  |  |  | [rs192456466](http://www.ncbi.nlm.nih.gov/projects/SNP/snp_ref.cgi?rs=rs192456466) | A>G | Validated | G=0.002/5 |  |
| hsa-mir-622 / *KRT18* (pseudogene) | 3p | x |  |  |  | [rs111371406](http://www.ncbi.nlm.nih.gov/projects/SNP/snp_ref.cgi?rs=rs111371406) | T>C | Validated | C=0.003/7 |  |
| hsa-mir-628 / *CCPG1, DYX1C1-CCPG1* | 3p | x |  |  |  | [rs148138194](http://www.ncbi.nlm.nih.gov/projects/SNP/snp_ref.cgi?rs=rs148138194) | TTCGA>- | Validated | NA |  |
| hsa-mir-640 / *GATAD2A* | 3p |  |  | x |  | [rs111726405](http://www.ncbi.nlm.nih.gov/projects/SNP/snp_ref.cgi?rs=rs111726405) | A>G | Unknown | NA |  |
| hsa-mir-656 / intergenic | 3p |  | x |  |  | [rs185468172](http://www.ncbi.nlm.nih.gov/projects/SNP/snp_ref.cgi?rs=rs185468172) | T>C | Validated | C=0.000/1 |  |
| hsa-mir-936 / *COL17A1* | 5p |  |  | x |  | rs140657237 | A>G | Validated | T=0.005/11 |  |
| hsa-mir-941-1 / *DNAJC5* | 3p | x |  |  |  | [rs112027130](http://www.ncbi.nlm.nih.gov/projects/SNP/snp_ref.cgi?rs=rs112027130) | C>A | Unknown | NA |  |
| hsa-mir-1178 / *CIT* | 5p | x |  |  |  | [rs74614893](http://www.ncbi.nlm.nih.gov/projects/SNP/snp_ref.cgi?rs=rs74614893) | G>A | Validated | A=0.011/23 |  |
| hsa-mir-1225 / *PKD1* | 3p |  | x |  |  | [CS993238](http://www.ncbi.nlm.nih.gov/projects/SNP/snp_ref.cgi?rs=CS993238) | .>. | - |  |  |
| hsa-mir-1227 / *PLEKHJ1* | 3p |  |  | x |  | [rs139405773](http://www.ncbi.nlm.nih.gov/projects/SNP/snp_ref.cgi?rs=rs139405773) | A>G | Validated | A=0.001/2 |  |
| hsa-mir-1233-1 / *GOLGA8A* | 5p |  |  |  | x | [rs347882](http://www.ncbi.nlm.nih.gov/projects/SNP/snp_ref.cgi?rs=rs347882) | C>G | Validated | NA |  |
| hsa-mir-1255b-1 / intergenic | 5p |  | x |  |  | [rs6841938](http://www.ncbi.nlm.nih.gov/projects/SNP/snp_ref.cgi?rs=rs6841938) | A>G | Validated | A=0.093/202 |  |
| hsa-mir-1255b-2 / *DCAF6* | 3p |  | x |  |  | [rs79639536](http://www.ncbi.nlm.nih.gov/projects/SNP/snp_ref.cgi?rs=rs79639536) | A>G | Validated | G=0.019/42 |  |
| hsa-mir-1262 / *WLS; RP11-518D3.1, GNG12-AS1* - antisense | 5p |  |  |  | x | [rs147113488](http://www.ncbi.nlm.nih.gov/projects/SNP/snp_ref.cgi?rs=rs147113488) | C>T | Validated | T=0.006/14 |  |
| hsa-mir-1273c / *TIAM2* | 5p |  |  | x |  | [rs111252952](http://www.ncbi.nlm.nih.gov/projects/SNP/snp_ref.cgi?rs=rs111252952) | ->T | Unknown | NA |  |
| hsa-mir-1290 / *ALDH4A1, RP13-279N23.2* | 3p | x |  |  |  | [rs147869275](http://www.ncbi.nlm.nih.gov/projects/SNP/snp_ref.cgi?rs=rs147869275) | A>G | Validated | G=0.006/12 |  |
| hsa-mir-1324 / intergenic | 3p |  |  |  | x  TNP | [rs112915509](http://www.ncbi.nlm.nih.gov/projects/SNP/snp_ref.cgi?rs=rs112915509) | A>G | Unknown | NA |  |
|  |  |  |  | x  TNP |  | [rs10155043](http://www.ncbi.nlm.nih.gov/projects/SNP/snp_ref.cgi?rs=rs10155043) | C>T | Unknown | NA |  |
|  |  |  |  |  |  | rs79101433* | A>G | Validated | NA |  |
| hsa-mir-1470 / *WIZ* | 5p |  |  |  | x | [rs113568246](http://www.ncbi.nlm.nih.gov/projects/SNP/snp_ref.cgi?rs=rs113568246) | G>A | Unknown | NA |  |
| hsa-mir-1910 / *C16orf74* | 5p |  |  | x |  | [rs183226620](http://www.ncbi.nlm.nih.gov/projects/SNP/snp_ref.cgi?rs=rs183226620) | A>G | Validated | G=0.001/2 |  |
| hsa-mir-2114 / intergenic | 5p |  |  |  | x | [rs188839575](http://www.ncbi.nlm.nih.gov/projects/SNP/snp_ref.cgi?rs=rs188839575) | G>A | Validated | A=0.010/16 |  |
| hsa-mir-3118-4 / intergenic | 3p |  |  |  | x | [rs1814008](http://www.ncbi.nlm.nih.gov/projects/SNP/snp_ref.cgi?rs=rs1814008) | C>T | Validated | NA |  |
| hsa-mir-3118-5 / intergenic | 3p |  |  |  | x | [rs141566892](http://www.ncbi.nlm.nih.gov/projects/SNP/snp_ref.cgi?rs=rs141566892) | C>T | Validated | NA |  |
| hsa-mir-3118-6 / intergenic | 3p |  |  |  | x | [rs1814008](http://www.ncbi.nlm.nih.gov/projects/SNP/snp_ref.cgi?rs=rs1814008) | C>T | Validated | NA |  |
| hsa-mir-3119-1 / *METTL11B* - antisense | 5p |  |  |  | x | [rs58602811](http://www.ncbi.nlm.nih.gov/projects/SNP/snp_ref.cgi?rs=rs58602811) | G>T | Validated | G=0.014/30 |  |
| hsa-mir-3125 / *TRIB2* | 5p |  |  |  |  | rs72536414* | ->A | Unknown | NA |  |
|  |  | x  TNP |  |  |  | [rs5829384](http://www.ncbi.nlm.nih.gov/projects/SNP/snp_ref.cgi?rs=rs5829384) | ->A | Validated | -=0.281/612 |  |
|  |  |  | x  TNP |  |  | [rs33977954](http://www.ncbi.nlm.nih.gov/projects/SNP/snp_ref.cgi?rs=rs33977954) | ->A | Validated | NA |  |
| hsa-mir-3155a / *PFKFB3* | 3p | x |  |  |  | [rs140190031](http://www.ncbi.nlm.nih.gov/projects/SNP/snp_ref.cgi?rs=rs140190031) | G>C | Validated | C=0.003/6 |  |
| hsa-mir-3158-2 / *DPCD* | 5p |  |  |  | x | [rs112037539](http://www.ncbi.nlm.nih.gov/projects/SNP/snp_ref.cgi?rs=rs112037539) | G>A | Validated | A=0.000/1 |  |
| hsa-mir-3161 / *PTPRJ* | 5p |  |  | x |  | [rs190080389](http://www.ncbi.nlm.nih.gov/projects/SNP/snp_ref.cgi?rs=rs190080389) | T>C | Validated | C=0.001/3 |  |
| hsa-mir-3170 / *FARP1* | 5p |  |  | x |  | [rs147240207](http://www.ncbi.nlm.nih.gov/projects/SNP/snp_ref.cgi?rs=rs147240207) | T>C | Validated | C=0.005/10 |  |
| hsa-mir-3177 / *MAPK8IP3* | 3p |  |  |  | x | [rs28575325](http://www.ncbi.nlm.nih.gov/projects/SNP/snp_ref.cgi?rs=rs28575325) | G>A | Validated | NA |  |
| hsa-mir-3177 / *MAPK8IP3* | 3p | x |  |  |  | [rs149035589](http://www.ncbi.nlm.nih.gov/projects/SNP/snp_ref.cgi?rs=rs149035589) | G>A | Validated | A=0.003/7 |  |
| hsa-mir-3196 / intergenic | 5p |  | x  DNP |  |  | [rs2273488](http://www.ncbi.nlm.nih.gov/projects/SNP/snp_ref.cgi?rs=rs2273488) | C>T | Unknown | NA |  |
|  |  |  |  |  |  | rs113297757 * | A>G | Validated | A=0.022/49 |  |
| hsa-mir-3199-1 / *TTC28-AS1* | 5p |  |  |  |  | [rs75321888](http://www.ncbi.nlm.nih.gov/projects/SNP/snp_ref.cgi?rs=rs75321888)* | ->G | Validated | NA |  |
|  |  | x  TNP |  |  |  | [rs75321888](http://www.ncbi.nlm.nih.gov/projects/SNP/snp_ref.cgi?rs=rs75321888) | ->G | Validated | NA |  |
|  |  |  | x  TNP |  |  | [rs78805657](http://www.ncbi.nlm.nih.gov/projects/SNP/snp_ref.cgi?rs=rs78805657) | G>T | Unknown | NA |  |
| hsa-mir-3615 / *SLC9A3R1; RP11-452I5.2* | 3p | x |  |  |  | [rs183452776](http://www.ncbi.nlm.nih.gov/projects/SNP/snp_ref.cgi?rs=rs183452776) | G>C | Validated | C=0.113/247 |  |
| hsa-mir-3674 / intergenic | 5p |  |  | x |  | [rs192186660](http://www.ncbi.nlm.nih.gov/projects/SNP/snp_ref.cgi?rs=rs192186660) | C>T | Validated | T=0.000/1 |  |
| hsa-mir-3679 / *MGAT5* | 5p | x |  |  |  | [rs6430498](http://www.ncbi.nlm.nih.gov/projects/SNP/snp_ref.cgi?rs=rs6430498) | G>A | Validated | A=0.367/799 |  |
| hsa-mir-3692 / *ZDHHC14* | 3p |  |  |  | x | [rs142312394](http://www.ncbi.nlm.nih.gov/projects/SNP/snp_ref.cgi?rs=rs142312394) | A>G | Validated | G=0.001/3 |  |
| hsa-mir-3943 / *HECW1, HECW1-IT1* | 5p |  |  | x |  | [rs148859333](http://www.ncbi.nlm.nih.gov/projects/SNP/snp_ref.cgi?rs=rs148859333) | G>A | Validated | A=0.000/1 |  |
| hsa-mir-3973 / *LDLRAD3* | 3p |  | x |  |  | [rs183016698](http://www.ncbi.nlm.nih.gov/projects/SNP/snp_ref.cgi?rs=rs183016698) | G>A | Validated | A=0.000/1 |  |
| hsa-mir-4258 / *CKS1B* | 5p |  |  | x |  | [rs113220875](http://www.ncbi.nlm.nih.gov/projects/SNP/snp_ref.cgi?rs=rs113220875) | G>C | Unknown | NA |  |
| hsa-mir-4273 / *ZNF717* - antisense | 5p |  |  | x |  | [rs76333414](http://www.ncbi.nlm.nih.gov/projects/SNP/snp_ref.cgi?rs=rs76333414) | G>A | Unknown | NA |  |
| hsa-mir-4285 / *SH2B2* | 5p |  |  |  | x | [rs1293864](http://www.ncbi.nlm.nih.gov/projects/SNP/snp_ref.cgi?rs=rs1293864) | C>A | Validated | NA |  |
| hsa-mir-4297 / *EBF3* | 5p | x |  |  |  | [rs114362263](http://www.ncbi.nlm.nih.gov/projects/SNP/snp_ref.cgi?rs=rs114362263) | C>T | Validated | T=0.020/44 |  |
| hsa-mir-4474 / *MLLT3* | 3p | x |  |  |  | [rs74428911](http://www.ncbi.nlm.nih.gov/projects/SNP/snp_ref.cgi?rs=rs74428911) | G>T | Validated | T=0.013/29 |  |
| hsa-mir-4477b / intergenic | 3p | x |  |  |  | [rs34379288](http://www.ncbi.nlm.nih.gov/projects/SNP/snp_ref.cgi?rs=rs34379288) | G>T,A | Unknown | NA |  |
| hsa-mir-4482 / intergenic | 5p | x |  |  |  | [rs641071](http://www.ncbi.nlm.nih.gov/projects/SNP/snp_ref.cgi?rs=rs641071) | A>C | Validated | T=0.444/967 |  |
| hsa-mir-4520b / intergenic | 5p |  | x |  |  | [rs8078913](http://www.ncbi.nlm.nih.gov/projects/SNP/snp_ref.cgi?rs=rs8078913) | C>T | Validated | C=0.431/938 | Zheng et al., 2013 |
| hsa-mir-4524a / *ABCA6* | 5p |  |  |  |  | [rs186818349](http://www.ncbi.nlm.nih.gov/projects/SNP/snp_ref.cgi?rs=rs186818349)* | A>G | Validated | A=0.000/1 |  |
|  |  | x  DNP |  |  |  | rs60532713 | C>T | Validated | T=0.004/9 |  |
| hsa-mir-4532 / intergenic | 5p |  |  | x |  | [rs73177830](http://www.ncbi.nlm.nih.gov/projects/SNP/snp_ref.cgi?rs=rs73177830) | G>A | Validated | A=0.038/82 |  |
| hsa-mir-4535 / *FAM19A5* | 3p | x |  |  |  | [rs141741714](http://www.ncbi.nlm.nih.gov/projects/SNP/snp_ref.cgi?rs=rs141741714) | G>A | Validated | A=0.000/1 |  |
| hsa-mir-4649 / *AEBP1* | 3p |  | x |  |  | [rs113545244](http://www.ncbi.nlm.nih.gov/projects/SNP/snp_ref.cgi?rs=rs113545244) | A>T | Validated | NA |  |
| hsa-mir-4666b / *IL1RAPL1* | 5p |  |  | x |  | [rs140631495](http://www.ncbi.nlm.nih.gov/projects/SNP/snp_ref.cgi?rs=rs140631495) | C>G | Validated | G=0.002/4 |  |
| hsa-mir-4669 / *RXRA* | 3p | x  DNP |  |  |  | [rs111496711](http://www.ncbi.nlm.nih.gov/projects/SNP/snp_ref.cgi?rs=rs111496711) | ->A | Unknown | NA |  |
|  |  |  |  |  |  | rs35196866* | A>C | Validated | C=0.365/794 |  |
| hsa-mir-4671 / *SLC35F3* | 3p |  |  |  | x | [rs877722](http://www.ncbi.nlm.nih.gov/projects/SNP/snp_ref.cgi?rs=rs877722) | A>T | Validated | T=0.175/382 |  |
| hsa-mir-4675 / intergenic | 3p |  |  | x |  | [rs139755219](http://www.ncbi.nlm.nih.gov/projects/SNP/snp_ref.cgi?rs=rs139755219) | G>A | Validated | A=0.001/2 |  |
| hsa-mir-4684 / *EPHB2* | 3p |  |  |  | x | [rs191124214](http://www.ncbi.nlm.nih.gov/projects/SNP/snp_ref.cgi?rs=rs191124214) | G>A | Validated | A=0.001/2 |  |
| hsa-mir-4724 / *RAB11FIP4* | 5p |  |  | x |  | [rs144274743](http://www.ncbi.nlm.nih.gov/projects/SNP/snp_ref.cgi?rs=rs144274743) | G>A | Validated | A=0.000/1 |  |
| hsa-mir-4745 / *PTBP1* | 5p |  |  |  |  | rs147466582* | C>T | Validated | T=0.001/3 |  |
|  |  |  |  | x  DNP |  | [rs139864698](http://www.ncbi.nlm.nih.gov/projects/SNP/snp_ref.cgi?rs=rs139864698) | G>A | Validated | A=0.003/6 |  |
| hsa-mir-4749 / *PTOV1* | 3p |  |  | x  DNP |  | [rs147943327](http://www.ncbi.nlm.nih.gov/projects/SNP/snp_ref.cgi?rs=rs147943327) | C>T | Validated | T=0.005/10 |  |
|  |  |  |  |  |  | rs148982635* | A>G | Validated | A=0.000/1 |  |
| hsa-mir-4754 / *RPS5* - antisense | 5p |  |  |  | x | [rs975947](http://www.ncbi.nlm.nih.gov/projects/SNP/snp_ref.cgi?rs=rs975947) | A>C | Validated | T=0.363/791 |  |
| hsa-mir-4762 / *ATXN10* | 5p |  | x |  |  | [rs41524547](http://www.ncbi.nlm.nih.gov/projects/SNP/snp_ref.cgi?rs=rs41524547) | C>G | Validated | G=0.013/29 |  |
| hsa-mir-4762 / *ATXN10* | 3p |  |  |  | x | [rs138025546](http://www.ncbi.nlm.nih.gov/projects/SNP/snp_ref.cgi?rs=rs138025546) | G>A | Validated | A=0.003/6 |  |
| hsa-mir-4783 / *PROC* | 3p |  | x |  |  | [rs117721121](http://www.ncbi.nlm.nih.gov/projects/SNP/snp_ref.cgi?rs=rs117721121) | G>A | Validated | A=0.005/11 |  |
| hsa-mir-4797 / *DLG1* | 3p |  |  |  | x  DNP | rs3836429 | ->A | Validated | -=0.390/849 |  |
|  |  |  |  |  |  | rs3836429 * | ->A | Validated | -=0.390/849 |  |
| hsa-mir-4797 / *DLG1* | 3p | x |  |  |  | [rs140219060](http://www.ncbi.nlm.nih.gov/projects/SNP/snp_ref.cgi?rs=rs140219060) | A>G | Validated | A=0.005/10 |  |
| hsa-mir-4798 / *SORCS2* | 3p |  |  |  | x | [rs114771990](http://www.ncbi.nlm.nih.gov/projects/SNP/snp_ref.cgi?rs=rs114771990) | C>T | Validated | T=0.018/40 |  |
| hsa-mir-5007 / intergenic | 3p | x |  |  |  | [rs190647137](http://www.ncbi.nlm.nih.gov/projects/SNP/snp_ref.cgi?rs=rs190647137) | T>A | Validated | A=0.001/2 |  |
| hsa-mir-5186 / intergenic | 3p | x |  |  |  | [rs9842591](http://www.ncbi.nlm.nih.gov/projects/SNP/snp_ref.cgi?rs=rs9842591) | A>C | Validated | A=0.493/1074 |  |
| hsa-mir-5585 / *TMEM39B* | 3p |  |  |  | x | [rs183961911](http://www.ncbi.nlm.nih.gov/projects/SNP/snp_ref.cgi?rs=rs183961911) | C>T | Validated | T=0.000/1 |  |
| hsa-mir-5702 / intergenic | 3p |  | x |  |  | [rs74949342](http://www.ncbi.nlm.nih.gov/projects/SNP/snp_ref.cgi?rs=rs74949342) | C>G | Validated | G=0.003/6 |  |
| hsa-mir-6723 / *RP5-857K21.4; RP5-857K21.6* - antisense | 5p |  | x |  |  | rs2000095 | C>T | Validated | NA |  |

**Legend:** hsa-mir = human microRNA, CS = cleavage site, double nucleotide polymorphism, DNP = double nucleotide polymorphism, TNP = triple nucleotide polymorphism, * = SNP is a part of MNP, but does not overlap +/- 1 nt Drosha or Dicer cleavage site, - = not available.
